# Differential regulation of Effector and Regulatory T cell function by Blimp1

**DOI:** 10.1038/s41598-017-12171-3

**Published:** 2017-09-21

**Authors:** Rashmi Bankoti, Chihiro Ogawa, Truc Nguyen, Lena Emadi, Michael Couse, Soofia Salehi, Xuemo Fan, Deepti Dhall, Yizhou Wang, Jordan Brown, Vincent Funari, Jie Tang, Gislâine A. Martins

**Affiliations:** 1 Widjaja Foundation Inflammatory Bowel and Immunobiology Research Institute (IBIRI), Los Angeles, CA USA; 2 Fluidigm, South San Francisco, CA USA; 3 Dept of Pathology, Los Angeles, CA USA; 4 Genomics Core, Cedars-Sinai Medical Center (CSMC), Los Angeles, CA USA; 5 Departments of Medicine, Division of Gastroenterology, Los Angeles, CA USA; 6 Biomedical Sciences, Research Division of Immunology, CSMC, Los Angeles, CA USA

## Abstract

The transcriptional regulator Blimp1 plays crucial roles in controlling terminal differentiation in several lineages. In T cells, Blimp1 is expressed in both effector (Teff) and regulatory (Treg) cells, and mice with T cell-specific deletion of Blimp1 (Blimp1CKO mice) spontaneously develop severe intestinal inflammation, indicating a crucial role for Blimp1 in T cell homeostasis regulation. Blimp1 has been shown to function as a direct activator of the *Il10* gene and although its requirement for IL10 expression has been demonstrated in both Treg and Teff cells under inflammatory conditions, the intrinsic requirement of Blimp1 for homeostatic maintenance of these T cell subsets had not been investigated. Using mice with Foxp3^+^ Treg-cell specific deletion of Blimp1 and other approaches, here we show that Foxp3^+^ Treg cell-intrinsic expression of Blimp1 is required to control Treg and Teff cells homeostasis but, unexpectedly, it is dispensable to prevent development of severe spontaneous intestinal inflammation. In addition, we show that Blimp1 controls common and unique aspects of Treg and Teff cell function by differentially regulating gene expression in these T cell subsets. These findings document previously unappreciated aspects of Blimp1’s role in T cell biology and shed light on the intricate mechanisms regulating Treg and Teff cell function.

## Introduction

The transcription factor B-lymphocyte-induced maturation protein-1(Blimp1/PRDI-BFI) encoded by the *Prdm*1 gene plays crucial roles in the terminal differentiation of many different cell types^1,2^. Blimp1 is expressed in several hematopoietic lineages, including B and T lymphocytes, and myeloid cells^1^. Blimp1 was initially thought to function only as a repressor of gene expression. More recent studies^3,4^ however, indicate that Blimp1 can also function as an activator of gene expression. In T cells, Blimp1 regulates differentiation of follicular helper T cells^5^, IL10 expression in Foxp3^+^^3,6^ and Foxp3^−^^2,7–11^ T cells and CD8^+^ T cells effector/memory differentiation^12–14^, thus playing non-redundant roles in the function of both effector (Teff) and regulatory (Treg) cells.

Mice with T-cell-specific deletion of Blimp1 spontaneously develop chronic intestinal inflammation that resembles human inflammatory bowel disease (IBD)^6^, suggesting that Blimp1 could be a crucial regulator of T cell homeostasis. In line with that, Genome-wide-association studies GWAS have shown the association of polymorphisms in the *Prdm1* gene and IBD^15^ and other chronic inflammatory conditions in humans, including Rheumatoid Arthritis (RA) and Systemic Lupus Erythematosus (SLE)^16^.

Despite these associations and the dramatic phenotype of mice with T cell-specific Blimp1 deficiency, the mechanisms underlying Blimp1’s role in regulating T cell homeostasis are not fully understood and the intrinsic role of Blimp1 in regulating Teff and Treg cell function under homeostatic conditions has not been addressed *in vivo*.

In the present study, we have used a combination of different approaches to investigate Blimp1’s intrinsic roles in controlling Teff and Foxp3^+^ regulatory (Treg) cell gene expression and function *in vivo*. Our findings reveal that Blimp1 controls common and unique aspects of Teff and Treg cell function, regulating a substantial amount of unique target genes in each cell type. We also show that, differently from the severe inflammatory phenotype observed in mice with T cell-specific (CD4^cre^ or LCK^cre^-mediated) deletion of Blimp, Foxp3^+^ Treg cell-specific deletion of Blimp1 leads to only mild intestinal inflammation, highlining a Treg-cell independent role for Blimp1 in controlling Teff cell homeostasis.

## Results

### Homeostatic expression of Blimp1 is more frequent amongst Treg than Teff cells

**Figure 1:**
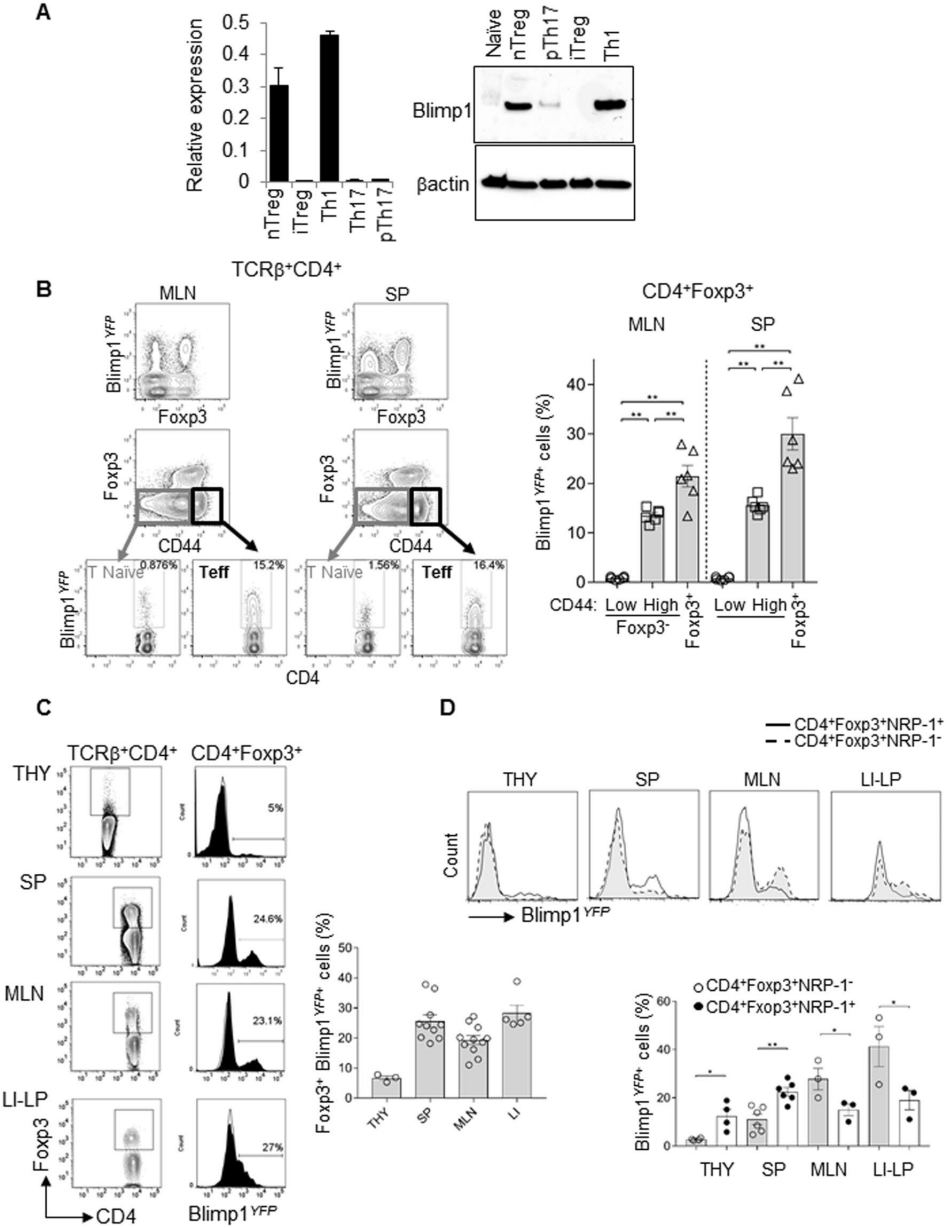
Blimp1 expression in CD4^+^ effector and Foxp3^+^ Treg cells under homeostatic conditions. (**A**) Quantitative Real time-PCR (qRT-PCR) analysis *Prdm1* (*Blimp1*) mRNA (relative to β2-microglobulin) (left) and Western blotting (right) of CD4^+^ Foxp3^*GFP*+^ Treg (nTreg) sorted from Ctrl Foxp3^*GFP*^ mice or *in vitro* differentiated Treg (iTreg,), Th1, Th17 or pathogenic (p) Th17 cells differentiated from naïve cells from the same mice (C57BL/6). (N = 3 mice/group, qPCR and N = 2 mice/sample, Western blotting). (**B**) FACS plot shows *Prdm1* mRNA expression (as reported by YFP, Blimp1^*YFP*^) among peripheral Treg (Foxp3^+^ as determined by intracellular staining of Foxp3 protein), effector (Foxp3^−^ CD44^high^) and naïve (Foxp3^−^ CD44^low^) TCRβ^+^ CD4^+^ cells in mesenteric lymph nodes (MLN, left) and spleen (SP, right). Bar graph shows the average percentage of Blimp1^*YFP*+^ cells among TCRβ^+^ CD4^+^ Foxp3^+^, TCRβ^+^ CD4^+^ Foxp3^−^ CD44^high^ or TCRβ^+^ CD4^+^ Foxp3^−^ CD44^low^ cells. (**C**) FACS plots and histograms overlay show percent of Blimp1^*YFP*+^ cells in gated TCRβ^+^ CD4^+^ Foxp3^+^ cells from thymus (THY), spleen (SP), mesenteric lymph nodes (MLN) and large (LI) intestines lamina propria (LP) from control (open histogram) and Blimp1^*YFP*^ (filled histogram) mice. Gating of Foxp3^+^ cells (as determined by intracellular staining of Foxp3 protein) is shown in FACS plots on the left. Cumulative data from several mice is shown on graph (right). (**D**) FACS histograms show analysis of Blimp1^*YFP*^ expression in gated TCRβ^+^ CD4^+^ Foxp3^+^ Neuropilin-1 (Nrp-1)^+^ (full line, empty histograms) and TCRβ^+^ CD4^+^ Foxp3^+^ Nrp-1^−^ (dashed line, filled histograms) cells in THY, SP, MLN and LI-LP from Blimp1^*YFP*^ mice. Lower panel shows percent of Blimp1^*YFP*+^ cells in CD4^+^ Foxp3^+^ Nrp-1^+^ (filled circles) and CD4^+^ Foxp3^+^ Nrp-1^−^ (open circles) cells iin different organs. For all graphs, each symbol represents one mouse. (N = 2–11 mice/group). *P < 0.05 and **P < 0.01, one-way ANOVA (**B**) and paired *t*- test (**C**). Error bars indicate SEM.

To begin to evaluate Blimp1’s requirement for Foxp3^+^ Treg and Foxp3^−^ Teff cells function, we first confirmed Blimp1 mRNA and protein expression in different Teff and Foxp3^+^ Treg cell subsets. We evaluated *in vitro*-derived inducible (i) Foxp3^+^ T cells and naturally occurring (n) Foxp3^+^ Treg cells side-by-side with *in vitro* derived Th1 and Th17 cells, which we have previously reported to express high and low levels of Blimp1, respectively^17^. For these experiments, we used Th17 cells differentiated under standard conditions (addition of recombinant IL23 and TGFβ) which we^17^ and others^7,8^ have previously reported to express very little to none Blimp. We have also included Th17 cells differentiated under pathogenic conditions (i.e. presence of added rMuIL23 and neutralizing anti-TGFβ antibodies), which were previously reported by Jain *et al*.^18^, to have substantial expression of Blimp1. As expected, we found that nTreg cells expressed high levels of Blimp1 mRNA and protein, which were slightly reduced in comparison to Th1 cells, but significantly higher than Th17 cells and pathogenic Th17 cells (Fig. 1A). Foxp3^+^ *in vitro*-derived iTreg cells on the other hand, expressed very little Blimp1 in comparison with nTreg and effector Th1 cells. In fact, Blimp1mRNA expression in iTreg was as low as observed in Th17 cells and Blimp1 protein was undetectable by western blotting (Fig. 1A), most likely as a result of TGFβ presence in the cultures, which can inhibit Blimp1 expression in T cells^17^. It is also possible that the differential expression of Blimp1 in Foxp3^+^ n and iTreg subsets could be due to different levels of IRF4 expression, as IRF4 has been previously shown to serve as direct activator of Blimp1 expression in Treg cells^3^ but these possibilities remain to be tested.

Analysis of Blimp1 expression under homeostatic conditions by Flow cytometry in cells from Blimp1-YFP reporter mice further confirmed previous reports^3,6,19^ of substantial expression of Blimp1 in peripheral CD4^+^ Foxp3^+^ Treg and CD44^high^ Teff cells in comparison to CD4^+^ CD44^low^ naïve T cells (Fig. 1B). Direct comparison of Blimp1 (YFP) expression in Foxp3^+^ Treg and Foxp3^−^ CD44^high^ Teff cells revealed that the frequency of Blimp1-expressing cells is significantly higher in Treg than in Teff (Foxp3^−^ CD44^high^ cells) in both spleen (SP) and mesenteric lymph nodes (MLN) (Fig. 1B), suggesting a stronger requirement for Blimp1 in Treg than in Teff cells in peripheral lymphoid tissues. Thus, under homeostatic conditions *in vivo*, Blimp1 expression is more frequent in Foxp3^+^ Treg cells than in Foxp3^−^ Teff cells.

### Variable expression of Blimp1 at the single cell and population levels in Foxp3^+^ Treg cells

Although previous studies^3,6^ have reported evaluation of Blimp1 expression in Treg cells, the simultaneous expression of Blimp1 and Foxp3 has not been systematically evaluated at the single cells level under homeostatic conditions before. Thus, we next assessed Blimp1 expression in Foxp3^+^ Treg cells at different sites, including lymphoid and non-lymphoid tissues. We found that the frequency of Blimp1-expressing Foxp3^+^ Treg cells is higher in the periphery than in the thymus (Fig. 1C). Blimp1-expressing Foxp3^+^ peripheral Treg were predominant in the intestinal lamina propria, especially in the large intestines. In line with previous observations that Blimp1 is preferentially expressed in activated/effector T cells^6^, Blimp1-expressing Foxp3^+^ Treg cells also from the SP and MLN expressed high levels of the activation marker CD44 (Suppl. Figure 1A). Evaluation of Neuropilin-1 (NRP-1) expression, which has been shown by some^20,21^ but not all studies^22^ to be preferentially associated with Thymus-developed (t) Treg cells, revealed that although both NRP1^+^ and NRP1^−^ Foxp3^+^ peripheral Treg cells expressed Blimp1, the frequency of Blimp1expressors in these subsets varied at different sites. The frequency of NRP-1^+^ Foxp3^+^ Treg cells expressing Blimp1 was significantly higher than their counterpart (NRP-1^−^ Foxp3^+^) in both, THY and SP, whereas in the MLN and LI-LP, the frequency of Blimp1-expressing cells was significantly higher amongst NRP1^-^ Foxp3^+^ Treg cells than in the NRP-1^−^ cells (Fig. 1D). Thus, Nrp1^+^ and Nrp1^−^ Foxp3^+^ Treg cells express Blimp1. This observation, together with our finding that Blimp1 expression can be also detected in thymus, albeit in a small percentage of Foxp3^+^ cells, suggests that Blimp1 is expressed in both thymic and peripherally-originated Foxp3^+^ Treg cells.

As expected from the fact that Blimp1 is required for IL10 expression by both Foxp3^+^ and Foxp3^−^ Teff cells, analysis of Blimp1 expression in Foxp3^*GFP*−^ IL10^*Thy1.1*+^ cells^23^ isolated from IL10-reporter^24^ crossed to Foxp3^*GFP*^mice showed similar levels of *Prdm1* mRNA in IL10-expressing Foxp3^+^ and Foxp3^−^ CD4^+^ T cells (Suppl. Figure 1B). Thus, except for *in vitro*-derived iTreg cells, all Foxp3^+^ Treg subsets have measurable Blimp1 expression under homeostatic conditions, albeit expression levels vary at different locations.

To gain further insight into the functional characteristics of Blimp1-expressing peripheral Treg cells, we simultaneously analyzed the expression of Blimp1 and the regulatory cytokines IL10 and TGFβ mRNA at the single cell level in *in vitro* stimulated Foxp3^+^ Treg cells. We sort purified CD4^+^ CD25^high^ cells from the spleen and lymph nodes from naïve mice and stimulated the cells *in vitro* with PMA and ionomycin to evaluate cytokine production upon TCR stimulation. Once stimulated, cells were then single sorted and submitted to quantitative real time PCR analysis using Fluidigm Dynamic arrays, which allowed simultaneous measurement of the expression of *Foxp3, Prdm1* (*Blimp1*), *Il10*, *Tgfb* and four different housekeeping genes (*B2m, Hprt, Hsp90ab1*, and *Ubc*) mRNA at the single cell level. These generated expression data for 96 single cells. After excluding failed reactions and cells that did not have expression of at least 3 of out of four housekeeping genes, we retained expression data for 71 single cells for data analysis.

**Figure 2:**
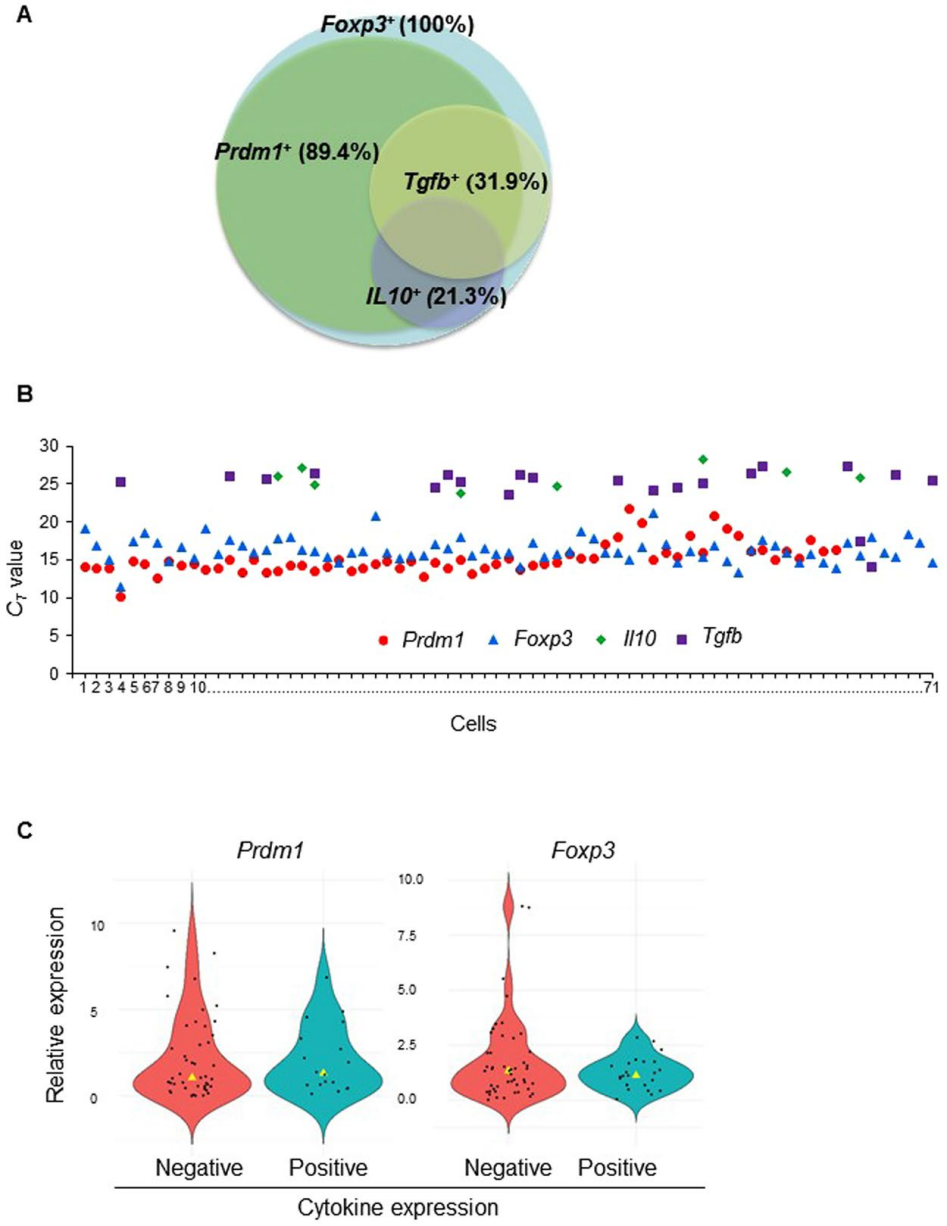
Single cell qRT-PCR analysis of *Prdm1* (*Blimp1*) expression in Treg cells. (**A**) Venn diagram showing frequency of *Foxp3*, *Prdm1*, *Il10* and *Tgfb*-expressing cells in all analyzed CD4^+^ CD25^high^ T cells single cells. (**B**) *C*_*t*_ values of *Foxp3*, *Prdm1*, *Tgfb* and *Il10* in all CD4^+^ CD25^high^ T cells analyzed. Each symbol represents one cell. (**C**) Violin plots showing relative expression of *Prdm1* (left) and *Foxp3* (right) in cells that expressed (positive) or lacked (negative) cytokines (*Tgfb* and or *Il10*) expression. Each circle indicates one cell. Yellow triangles indicate median.

Our analysis revealed that 100% of the cells expressed high levels of Foxp3 mRNA, as expected from our sorting strategy. However, differently from the observed at steady-state conditions, in which approximately 10–30% of Foxp3^+^ Treg cells express *Prdm1* mRNA (as reported by YFP expression) (Fig. 1B,C) the majority (89.4%) of TCR-stimulated Foxp3^+^ cells expressed measurable amounts of *Prdm1* mRNA in our single cell PCR analysis (Fig. 2A,B). This observation was also confirmed by analysis of Blimp1 expression by qRT-PCR (using different *Prdm1* primer sets) in bulk Foxp3^+^ and Foxp3^+^ BlimpYFP^-^ Treg cells which showed increased Blimp1 expression upon *in vitro* TCR stimulation (Suppl. Figure 2A). Expression of *Tgfb* and *Il10*, which have been previously associated with Treg cell function was detected in a smaller proportion of the cells: 31.9% of the cells were *Tgfb* ^+^ and 21.3% were *Il10* ^+^ (Fig. 2A), with 7.0% of the cells simultaneously expressing both *Il10* and *Tgfb*, suggesting that effector Treg cells might be composed of different effector subsets, with differential capability for cytokine production.

Although the majority of *Il10* and/or *Tgfb*-expressing cells also expressed *Prdm1*, 23.8% of all *Tgfb* ^+^ cells (7.1% of total) were *Prdm1* ^−^ and 10% of all *Il10* ^+^ cells (1.4% of the total Foxp3^+^ cells) were *Prdm1* ^−^ (Fig. 2A). Further analysis showed that expression of both *Foxp3* and *Prdm1* were highly variable (Fig. 2B) and only weakly correlated at the single cell level (Suppl. Figure 2B). Despite the variation in the levels of *Prdm1* mRNA expression in the Foxp3^+^ Treg cells, and the fact that most cytokine-expressing cells were *Prdm1* ^+^, we found no correlation between the levels of *Prdm1* and *Tgfb* or *Il10* expression (Suppl. Figure 2B). Moreover, *Prdm1* and *Foxp3* mRNA expression levels were not significantly different amongst *Il10* and or *Tgfb*-expressing cells (Fig. 2C). Thus, expression of *Prdm1* mRNA is variable and it does not fully correlate with expression of the regulatory cytokines *Il10* and *Tgfb* mRNA at the single cell level in Foxp3^+^ Treg cells. Future single cells analysis studies focusing on the simultaneous evaluation of protein levels of Blimp1 and Treg-specific effector molecules should help to further understand the requirements of Blimp1 for Foxp3^+^ Treg cell function.

### Foxp3^+^ Treg cells-intrinsic expression of Blimp1 is required to control Treg cell homeostasis

**Figure 3:**
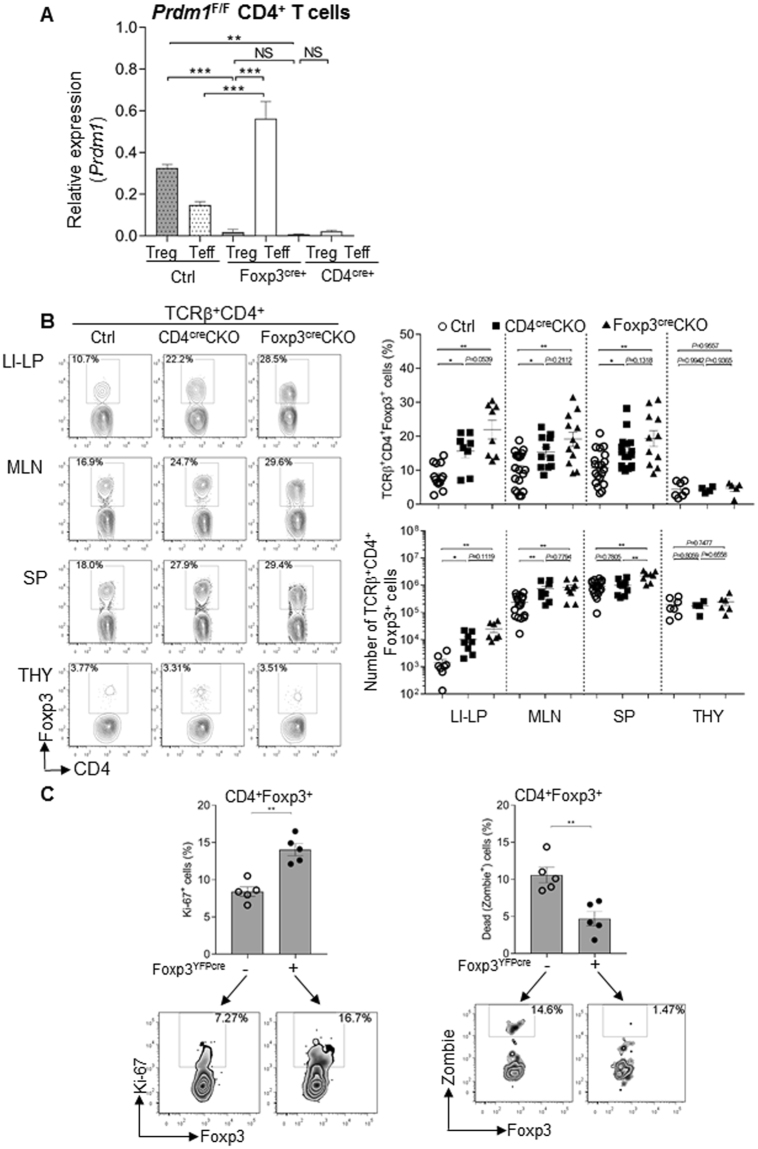
Increased Treg numbers in Blimp1 CKO mice is a cell-intrinsic effect associated with increased proliferation and decreased cell death. (**A**) Expression of *Prdm1* (Blimp) mRNA, (qRT-PCR, relative to β2Microglobulin) in sorted Treg (CD4^+^ Foxp3^GFP+^) and Teff (CD4^+^ Foxp3^−^ CD44^high^) cells from control (*Prdm1* ^F/F^), Foxp3^CRE^ CKO (*Prdm1* ^F/F^ Foxp3^cre+^), and CD4^CRE^ CKO (*Prdm1* ^F/F^ CD4^CRE+^) mice (**B**) Left panel; representative FACS plots of TCRβ^+^ CD4^+^ Foxp3^+^ cells (as determined by intracellular staining of Foxp3 protein) in large intestine lamina propria (LI-LP), mesenteric lymph nodes (MLN), spleen (SP) and thymus (THY) from control (Ctrl) or Blimp1 CKO (CD4^cre^CKO or Foxp3^cre^CKO) mice. Right graphs; percentage of Foxp3^+^ cells among TCRβ^+^ CD4^+^ cells (top) and absolute numbers of Foxp3^+^ cells (bottom) in LI-LP, MLN, SP and THY. Each symbol represents one mouse (N ≥ 4mice/group). Bars indicate average ± SEM. *P < 0.05, **P < 0.01, one-way ANOVA. (**C**) Top: graphs showing the average percentage ± SEM of Ki-67^+^ or Zombie (fixable viability dye)^+^ cells among CD4^+^ Foxp3^+^ (as determined by intracellular staining of Foxp3 protein) Foxp3^YFP-cre+^ or CD4^+^ Foxp3^+^ Foxp3^YFP-cre-^ cells from Foxp3^cre^CKO female mice. Each circle represents one mouse. Bottom: representative FACS plots showing analysis of Ki-67^+^ or Zombie^+^ cells (N = 5 mice). **P < 0.01, paired *t* -test. In (**A**) results representative of two different experiments; In (**B**) cell numbers were calculated based on the total number of cells obtained from each organ and population frequency determined by FACS analysis.

The results described above showing high expression of Blimp1 in Foxp3^+^ Treg cells under homeostatic conditions and further induction upon TCR stimulation, together with previous observations that Blimp1 is required for peripheral T cell homeostasis^6,25^ led us to enquire if intrinsic expression of Blimp1 in Foxp3^+^ Treg cells was required to maintain their homeostasis. To test this, we crossed our previously described *Prdm1* ^F/F^ mice^6^ with mice expressing knocked-in yellow fluorescent protein (YFP)/iCre-recombinase fusion protein from the *Foxp3* locus without disrupting expression of the endogenous *Foxp3* gene (Foxp3^YFP/cre^)^26^ to generate mice with Foxp3^+^ Treg cell-specific deletion of Blimp1 (Foxp3^cre^CKO). We first confirmed that deletion of *Prdm1* in these mice was Foxp3^+^ Treg cell-specific and found that in Foxp3CRECKO mice expression of *Prdm1* mRNA is significantly elevated in Teff cells as compared to Teff cells from Ctrl mice (Fig. 3A and Suppl. Figure 3A). We next evaluated and compared the frequency and absolute numbers of Foxp3^+^ Treg cells in the lymphoid organs and peripheral tissues of these mice with the observed in *Prdm1* ^F/F^ mice crossed with CD4^cre^ mice in which Blimp1 is deleted in all T cells^17,19^ (CD4^cre^CKO mice). Our results revealed that similarly to the observed in CD4^cre^CKO mice, Foxp3^cre^CKO mice had increased frequencies of Foxp3^+^ Treg cells in the periphery (Fig. 3B), indicating that the regulation of Treg cell homeostasis by Blimp1 is a cell-intrinsic effect. In addition, we found that in both CD4^cre^CKO and Foxp3^cre^CKO mice although the frequency of Foxp3^+^ Treg cells is increased in SP, MLN and LI-LP, it was not altered in the thymus, indicating that Blimp1 is required for the homeostasis of peripheral but not for thymus -derived Treg cells (Fig. 3B). As a control for CRE expression in these experiments we also evaluated the frequency and absolute numbers of Foxp3^+^ Treg cells in *Prdm1* ^*F/F*^ *, Prdm1* ^+/+^ CD4^cre^, and *Prdm1* ^+/+^ Foxp3^cre^ and found no significant differences amongst the three different genotypes (Suppl. Figure 3B), thus for all other experiments described here we used these mice interchangeably as controls.

To gain further insight into the mechanisms underlying cell intrinsic regulation of Treg cell homeostasis by Blimp1, we evaluated Foxp3^+^ cell proliferation and death in female *Prdm1* ^F/F^Foxp3^cre^mice, in which random X chromosome inactivation allowed for comparison of Blimp1-sufficient and Blimp1-deficient Foxp3^+^ Treg cells in the same animal. As shown in Fig. 3C, we found increased frequency of cells expressing the proliferation –associated antigen Ki-67 in Blimp1-deficient (Foxp3^YFP^/^*cre*^)^+^ Foxp3^+^ Treg cells in comparison to Blimp1-sufficient (Foxp3^YFP/*cre*^)^−^ Foxp3^+^ Treg cells in the same mice. Lack of Blimp1 was also associated with a decrease in the frequency of cell death amongst Foxp3^+^ Treg cells. Thus, Blimp1 controls Foxp3^+^ Treg cell homeostasis by regulating cell proliferation and survival in a cell-intrinsic manner.

### Expression of Blimp in Treg cells is required to control Teff cell homeostasis

**Figure 4:**
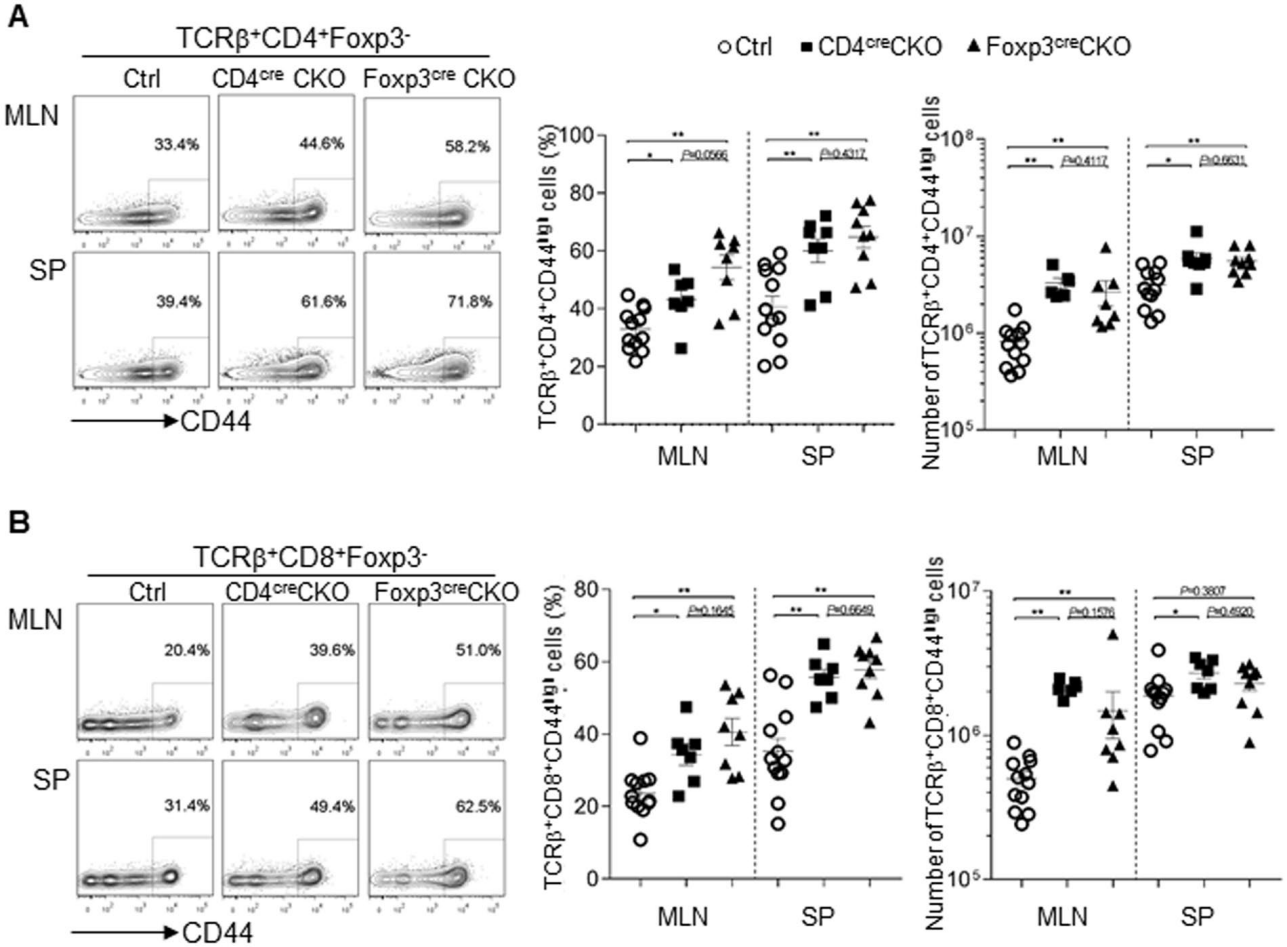
Treg-cell specific deletion of Blimp1 leads to Teff cells accumulation. (**A)** Representative FACS plots (left) show CD4^+^ (TCRβ^+^ CD4^+^ Foxp3^−^ CD44^high^) Teff cells in mesenteric lymphnodes (MLN) and spleen (SP) from control (Ctrl) or Blimp1 CKO (CD4^cre^CKO or Foxp3^cre^CKO) mice. Graphs show percentage (middle) and absolute number (right) of TCRβ^+^ CD4^+^ Foxp3^−^ CD44^high^ cells. (**B**) Representative FACS plots (left) of T effector CD8^+^ (TCRβ^+^ CD8^+^ Foxp3^−^ CD44^high^) cells in -MLN and SP from Ctrl, CD4^cre^CKO or Foxp3^cre^CKO mice. Graphs show percentage (middle) and absolute number (right, calculated as described on Fig. 3 legend) of TCRβ^+^ CD8^+^ Foxp3^−^ CD44^high^ cells. Each symbol represents one mouse (N ≥ 6 mice/group). Bars indicate average ± SEM. *P < 0.05, **P < 0.01, one-way ANOVA.

Previous studies established the role of Foxp3^+^ Treg cells in controlling Teff cell homeostasis^27,28^. Since Foxp3^+^ Treg cells-specific deletion of Blimp1 led to increased number of Treg cells, we next asked if this would interfere with Teff cell homeostasis. As shown in Fig. 4, mice with Foxp3^−^ specific deletion of Blimp1 had significantly higher frequencies and numbers of CD4^+^ (Fig. 4A) and CD8^+^ (Fig. 4B) (CD44^high^) Teff cells in the periphery. Of note, the accumulation of Teff cells in Foxp3^cre^CKO mice was similar to the observed in CD4^cre^CKO mice. Thus, expression of Blimp1 in Foxp3^+^ Treg cells is required for control of Teff cell homeostasis.

### Foxp3^+^ Treg cell-specific deletion of Blimp1 is not sufficient to cause severe chronic intestinal inflammation

**Figure 5:**
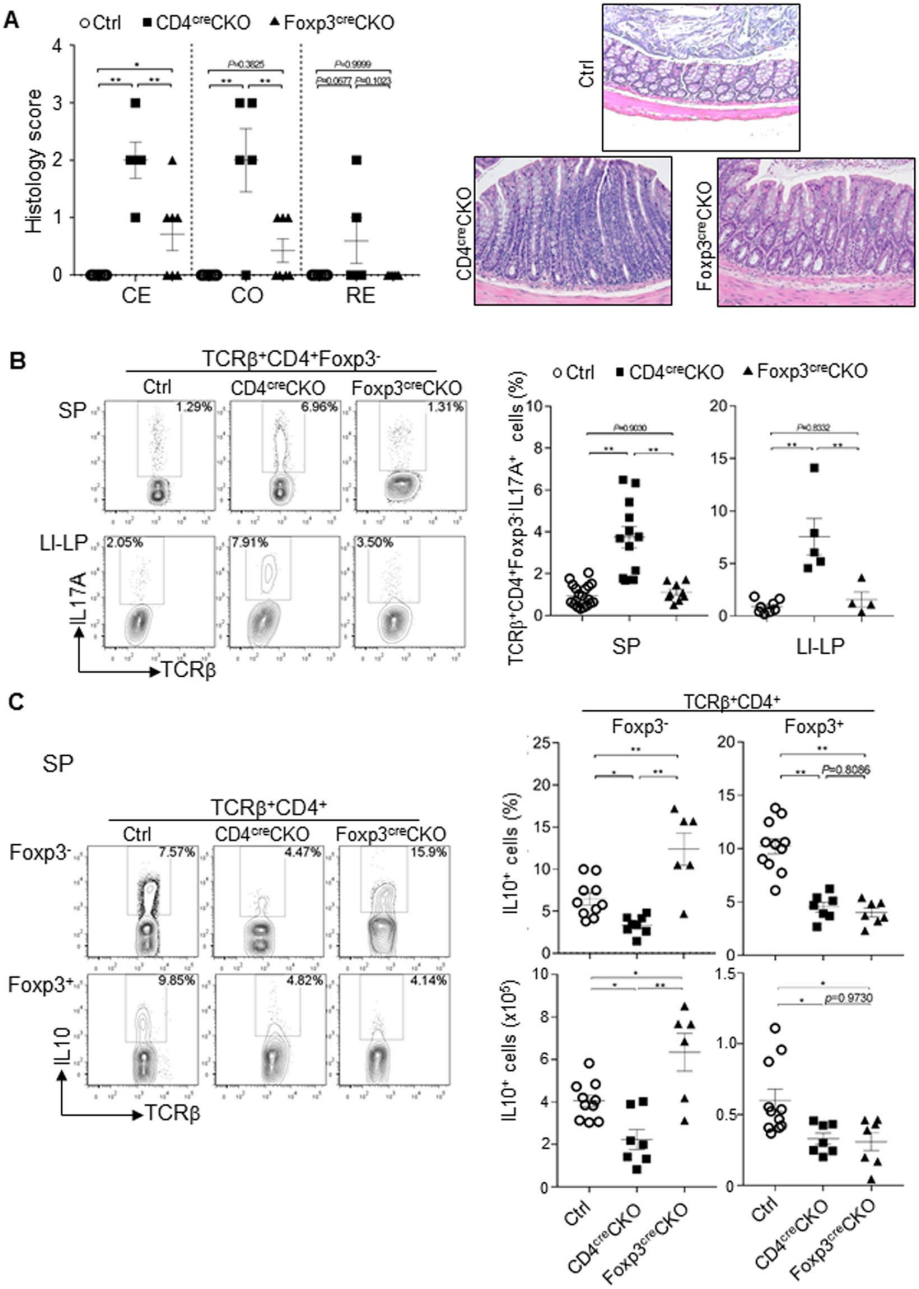
Treg-cell intrinsic deletion of Blimp1 leads to mild intestinal inflammation. (**A**) Left: scatter plots showing histology score of cecum (CE), colon (CO) and rectum (RE) among control (Ctrl), CD4^cre^CKO and Foxp3^cre^CKO mice. Each symbol represents one mouse (N ≥ 5 mice/group, Bars indicate average ± SEM. *P < 0.05, **P < 0.01, one-way ANOVA. Right: representative pictures (20X magnification) of histological sections of colon of mice in each group. (**B**) Representative FACS plots (left) and scatter plots (right) showing the frequency of IL17A expression in TCRβ^+^ CD4^+^ Foxp3^−^ cells in spleen (SP) and large intestine lamina propria (LI-LP) from Ctrl, CD4^cre^CKO or Foxp3^cre^CKO mice after stimulation. Right scatter plots: Each symbol represents one mouse (N ≥ 4 mice/group). Bars indicate average ± SEM. **P < 0.01, one-way ANOVA. (**C**) Representative FACS plots (left) and Scatter plots showing the frequency (right, top) or the total numbers (right, bottom) of IL10-producing cells in TCRβ^+^ CD4^+^ Foxp3^−^ or TCRβ^+^ CD4^+^ Foxp3^+^ cells in the SP from Ctrl, CD4^cre^CKO or Foxp3^cre^CKO mice. Each symbol represents one mouse (N ≤ 6 mice/group). Bars indicate average ± SEM. *P < 0.05, **P < 0.01, one-way ANOVA.

The results described above showing disruption of T cell homeostasis upon Foxp3^+^ Treg cell-specific deletion of Blimp1 together with previous observations that Blimp1 is required for IL10 production in Foxp3^+^ Treg cells^3,6^, led us to inquire if the previously described inflammatory phenotype of mice with T cell-specific deletion of Blimp1 could be recapitulated in mice with Foxp3^+^ Treg cells-specific deletion of Blimp1. We addressed that by evaluating development of spontaneous colitis in these two different mice lines. As shown in Fig. 5A, in comparison to CD4^cre^CKO, Foxp3^cre^CKO mice developed only mild intestinal inflammation. In addition, differently from the observed in cells from CD4^cre^CKO mice, and in line with previous findings that lack of Blimp1 leads to Teff cell-intrinsic induction of IL17A expression^7,17,29,30^, Foxp3^−^ CD4^+^ Teff cells from Foxp3^cre^CKO mice had unaltered production of the inflammatory cytokines IL17A (Fig. 5B) and similar results were observed on preliminary analysis of IFNγ expression (Suppl. Figure 4). As expected, Foxp3^+^ Treg cells from both CD4^cre^ and Foxp3^cre^ mice had reduced production of the anti-inflammatory cytokine IL10. The frequency and numbers of IL10-producing Teff cells from Foxp3^cre^CKO mice, on the other hand, were significantly increased in comparison to the observed in CD4^cre^CKO mice (Fig. 5C). Thus, Foxp3^+^ Treg-cell specific deletion of Blimp1 does not fully recapitulate the phenotype of mice with T cell specific deletion of Blimp1, developing only mild intestinal inflammation, associated with increased production of IL10 by CD4^+^ Teff cells.

### Blimp1 regulates unique and commonly shared genetic programs in Treg and Teff CD4^+^ T cells

**Figure 6:**
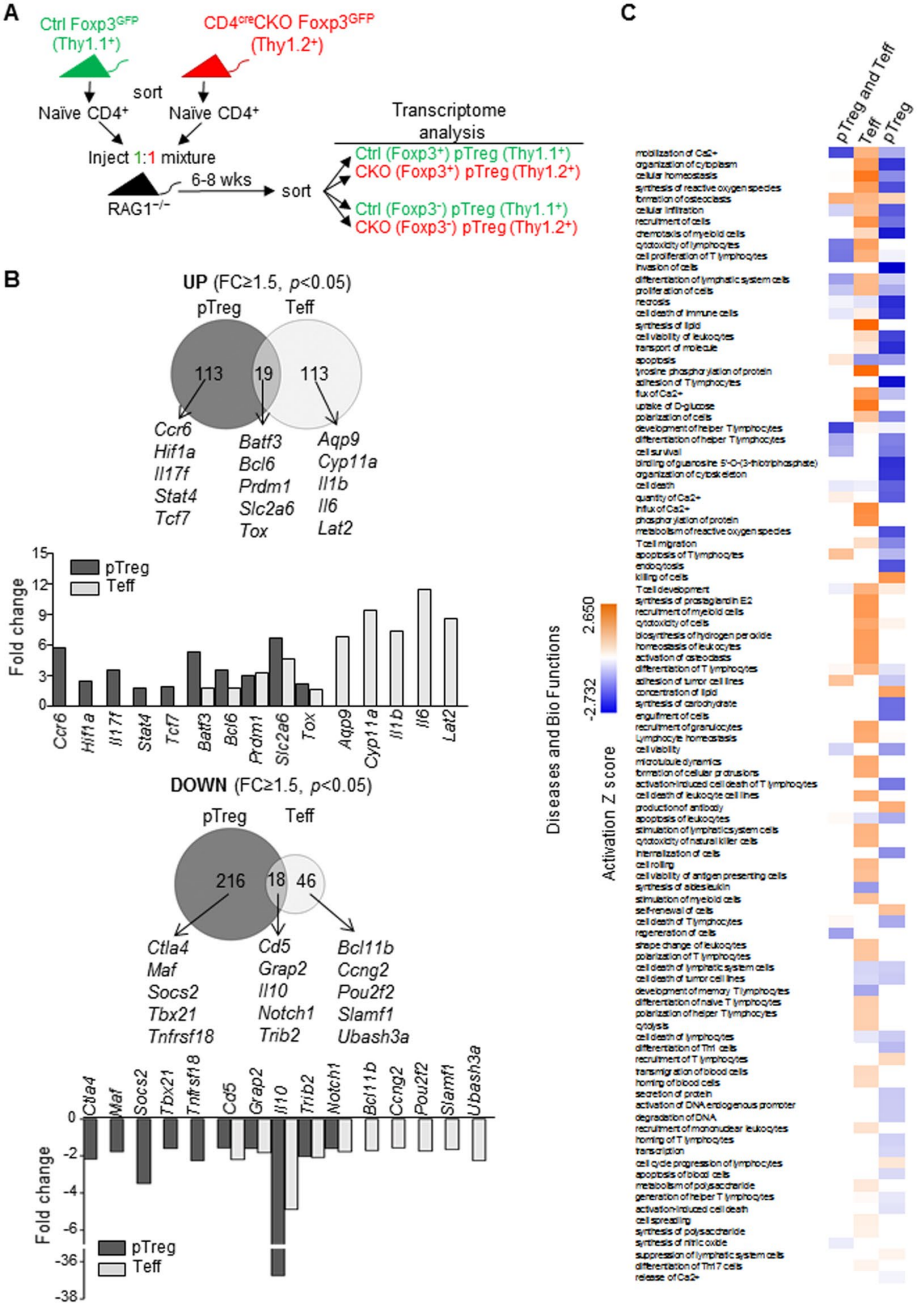
Blimp1 regulates unique and commonly shared genetic programs in CD4^+^ Treg and Teff cells. (**A**) Schematic representation of experimental approach used to obtain allotype-marked Blimp1-sufficient (Ctrl, Thy1.1^+^) and deficient (CKO, Thy1.2^+^) Teff (CD4^+^ Foxp3^−^) and Treg (CD4^+^ Foxp^3+^) cells differentiated in the same environment *in vivo*. (**B**) Venn diagrams representing the number of deferentially expressed (DE) genes upregulated (top) and downregulated (bottom) in pTreg and Teff cells (CD4^cre^CKO versus Ctrl) generated from Ctrl and CD4^cre^CKO CD4^+^ naive T cells co-injected into RAG1^−/−^ mice and used for mRNA microarray analysis. Three biological replicates were used for each sample. Bar graphs under Venn diagram show fold change for selected genes in each group. (**C**) IPA analysis of DE genes (1.5-fold at *P* < 0.05): shown are comparison analysis of diseases and bio functions between common DE genes in both Foxp3^*GFP*+^pTreg (pTreg_)_ and Foxp3^*GFP*−^ CD44^high^ (CD4^+^ Teff) cells, DE genes in CD4^+^ Teff cells only and in pTreg cells only (−log_10_ *p* value cut-off; 1.3).

To further explore the mechanisms underlying cell-intrinsic regulation of Treg and Teff cell function by Blimp1, we performed transcriptome analysis of CD4^+^ Blimp1-sufficient and deficient Foxp3^+^ Treg and Teff cells differentiated in the same environment *in vivo*. For these experiments, we took advantage of the fact that both Foxp3^+^ pTreg and Foxp3^−^ Teff cells can be generated from the same pool of CD4^+^ naïve cells upon transfer to RAG-deficient mice^22^. Thus, we transferred allotype-marked Blimp1-sufficient and deficient naïve (Foxp3^*GFP*−^ CD44^low^) CD4^+^ T cells into RAG1^−/−^ mice and then 6–8 weeks later recovered Blimp1-sufficient (Thy1.1^+^) and deficient (Thy1.2^+^) Teff (Foxp3^−^) and p Treg (Foxp3^+^) cells and performed RNA microarray analysis (Fig. 6A). This analysis revealed that in comparison to Blimp1-suficient cells, a total of 366 and 196 genes were differentially expressed in Blimp1-deficient pTreg and Teff cells respectively (FC ≥ 1.5, *p* < 0.05) (Suppl. Table 1). In line with the idea that Blimp1 functions mainly as a transcriptional repressor, from the 196 genes differentially expressed in Blimp1-deficient Teff cells, the majority (132) were up regulated and only 64 genes were down regulated. However, from the 366 genes differently expressed in Treg cells, the majority (234) was down-regulated, and only 132 genes were upregulated. Despite the similarity of Blimp1-regulated functional aspects in Teff and pTreg cells, we found that only 37 genes were commonly regulated by Blimp1 in these T cell subsets. From these, 19 genes were commonly up regulated in Teff and pTreg cells, including *Bcl6* and *Prdm1* which were previously shown to be directly repressed by Blimp1 in different cell types^5,31,32^ (Fig. 6B). Commonly down regulated genes (18) in Blimp1-deficient Teff and pTreg cells included the cytokine *Il10*, which was previously shown to be regulated by Blimp1 in both Foxp3^+^ and Foxp3^−^ cells and to be a direct target of Blimp1 in Foxp3^+^ Treg cells^3^.

Ingenuity pathway analysis (Fig. 6C) indicated several functional pathways specifically deregulated in pTreg or in Teff cells, and some of these pathways were common to both cell subsets. Commonly altered pathways included calcium mobilization, cytotoxicity and apoptosis, whereas pathways altered specifically in Teff cells included lipid synthesis and tyrosine phosphorylation. Pathways altered exclusively in pTreg cells included cytoplasm organization and synthesis of reactive oxygen (Fig. 6C). Thus, Blimp1 differentially regulate functional aspects of pTreg and Teff cells function by controlling unique and commonly shared genetic programs.

## Discussion

Our studies described here shed light on the mechanisms underlying the crucial role of the transcriptional factor Blimp1 in regulating T cell function. Using mice in which Blimp1 is specifically deleted in Foxp3^+^ cells and other approaches, we show that Foxp3^+^ T_reg_ cell-intrinsic expression of Blimp1 is required to control Treg and Teff cells homeostasis but is dispensable to prevent development of severe spontaneous intestinal inflammation. In addition, we show that Blimp1 regulates common and unique aspects of Foxp3^+^ Treg and Teff cell function by differentially controlling gene expression in these cells. These findings uncover previously unappreciated aspects of Blimp1’s role in T cell biology and shed light on the intricate mechanisms regulating Treg and Teff cell function.

Blimp1 is a transcriptional regulator that is crucial for terminal differentiation of several lineages. Its role in the immune system was first described in B cells (revised in^1^), but more recent studies also uncovered non-redundant roles for Blimp1 in myeloid cells^33,34^ and T lymphocytes^5–8,11–13,25,32^. Mice with T-cell specific deletion of Blimp1 (Blimp1CKO) spontaneously develop severe intestinal inflammation^6^ and can be potentially useful as an experimental model to study T cell-mediate intestinal pathology however, the mechanisms underlying disease development in these mice are not fully understood. Blimp1 functions as a direct activator of the *I* *l* *10* gene and although its requirement for IL10 expression has been demonstrated in both Treg and Teff cells under inflammatory conditions, the intrinsic requirement of Blimp1 for homeostatic maintenance of these different T cell subsets had not been investigated before.

Our observation that intestinal inflammation is significantly attenuated in mice with Treg-cell specific deletion of Blimp1 in comparison with mice lacking Blimp1 in all T cells strongly supports the idea that intrinsic expression of Blimp1 in Teff cell- is required for their homeostasis. This is further supported by the observation that the production of inflammatory cytokines such as IL17 and IFNγ, which are elevated in Teff cells from CD4^cre^CKO mice^7,17^ are unaltered in Foxp3^cre^CKO mice.

As expected from previous studies^3,6–8^ we found that expression of IL10 is significantly decreased in Treg cells from Foxp3^cre^CKO mice. Unexpectedly, however these mice also have a significant increase in the frequency and total numbers of IL10-producing Teff cells. The mechanisms underlying this increased IL10 expression remains to be elucidated. Our observation that Teff cells from the Foxp3^CRE^CKO mice also have a significant increase in Blimp1 expression (Fig. 3A) suggests a compensatory mechanism prompted by the IL10 defect in Treg cells and the inflammatory response associated with it, as observed in other inflammatory models in which an autoregulatory loop results in acquisition of Blimp1-dependent IL10 producing capabilities by inflammatory Teff cells^7,8,11,35^.

The observation that both CD4^cre^CKO and Foxp3^cre^CKO mice spontaneously develop intestinal inflammation would appear to be at odds with a recent study reporting that *Prdm* *1* ^F/F^ mice crossed with Distal LCK^cre^ transgenic mice do not show any inflammatory phenotype^18^. It is possible that these phenotypic differences could be due to the time of Blimp1 deletion during thymic development (early deletion in CD4^cre^ and Proximal LCK^cre^ versus late deletion in Distal LCK T cell). However, expression of Blimp1 in the thymus is very low^6^ and there is no evidence that Blimp1 could play a role in early T cell development. One caveat with the study mentioned above is that deletion of *Prdm1* in the Distal-LCK^cre^ transgenic mice reaches less than 60% of the total peripheral CD4^+^ T cells^18^, whereas deletion mediated by the Proximal LCK^cre^ and CD4^cre^ transgenes is substantially more efficient [^6^ and Fig. 3A]. Thus, one possibility is that *Prdm1* ^F/F^ Distal LCK^cre^ mice carry a larger number of Blimp1-sufficent Treg cells, which could suppress development of disease. This remains to be investigated and accurate comparison amongst these different models will also require co-housing of all mutant mice in the same mice facility, as the intestinal microbioma influences disease development and outcome in CD4^cre^CKO mice (R. Porritt and G. Martins, unpublished).

The effector mechanisms underlying development of mild, albeit detectable intestinal inflammation in Foxp3^cre^CKO mice are currently being investigated, but the fact that these mice develop any form of intestinal inflammation is surprising considering that expression of inflammatory cytokines such as IL17 and IFNγ, is not significantly elevated whereas expression of IL10 by non Treg cells is elevated. This could suggest that enhanced production of IL10 by non Treg cells is not sufficient to compensate for defective IL10 expression in Foxp3^+^ Treg cells, but this remains to be investigated.

Our transcriptome analysis, which revealed that the number of genes uniquely regulated by Blimp1 in either pTreg or Teff greatly surpass the number of genes commonly regulated genes further illustrates the ﻿differential intrinsic role of Blimp1 in these two different T cell subsets. Of note, we also found that in our system Blimp1 controls expression of a larger number of genes in Treg cells in comparison with Teff cells. The reason for this difference is not clear at the moment, but one possible explanation relates to the idea that Blimp1 could control gene expression on a dose-dependent manner^2^, which would imply regulation of different gene sets depending on Blimp1 concentration and availability in each cell type. We did find in our experiments that expression of Blimp1 was greater in Tregs in comparison to Teff (Figs 1 and 3). It is also likely that differential availability of co-factors in each cell type contribute to differential regulation of gene expression by Blimp1. Finally, one needs to consider the limitation of our system in which pTregs cells were generated under inflammatory conditions, which might differ from pTreg cells generated under homeostatic conditions. These possibilities remain to be tested.

In summary, our study provides evidence for a Teff-cell intrinsic, Treg cell-independent regulatory role for Blimp1 that goes beyond the regulation of IL10 expression. In addition, our findings support the notion that control of T cell function by Blimp1 is mediated by the regulation of a very broad range of unique and commonly shared genetic programs in both effector and regulatory T cells, which are most likely defined by the interaction of Blimp1 with different co-factors in a cell-specific manner.

## Materials and Methods

### Mice

C57BL/6*Prdm1* ^flox/floxCD4-Cre+^ (Blimp1CKO) and *Prdm1* ^+/+CD4-Cre+^ (Control, Ctrl) mice were previously described^6,19^. Mice expressing CRE recombinase and yellow fluorescent protein (YFP) under the control of the Foxp3 promoter, Foxp3^YFP-CRE^ (Foxp3^tm4 (YFP/cre)Ayr^) mice (Jackson laboratories) were crossed to C57BL/6*Prdm1* ^flox/flox^ to generate *Prdm1* ^flox/flox^/Foxp3^YFP-CRE^ mice. Blimp1CKO were bred to Foxp3^−^ IRES-GFPknock-in (Foxp3^*GFP*^) mice^36^ to generate Blimp1CKOFoxp3^*GFP*^mice. Mice bearing a BAC transgene encoding YFP under the control of Blimp1 regulatory elements (Blimp1^YFP^mice)^12,17^ were obtained from Eric Meffre (Yale University, New Haven, CT). All mice were bred and maintained in the CSMC SPF animal barrier facility and handled in accordance with the institutional guidelines. Mice were evaluated at 8–12 weeks of age. All experiments utilizing animals were approved by the CSMC Institutional Animal Care and Use Committee.

### Real-time quantitative PCR (qRT-PCR)

Total mRNA was isolated using RNAeasy kits (Qiagen, Valencia, CA) and reverse transcribed as previously described^17^. SYBR Green incorporation qRT- PCR was performed using FastStart SYBR Green Master mix (Roche, Pleasanton, CA) in the Realplex^2^ Mastercycler machine (Eppendorf, Hauppauge, NY). Primers sequences are as follows: β*2 m* FWR: GCTCGCGCTACTCTCTCTTT, β*2 m* REV: TCTGAATGCTCCACTTTTTCAA, *Prdm1* FWR: TTGAGATTGCTTGTGCTGCT, *Prdm1* REV: TCTCCAACCTGAAGGTCCAC, *Il10* FWR: AGCTGGACAACATACTGC, *Il10* REV: CTTGTAGACACCTTGGGTC. *Foxp3 FWR: CAGCTGCAGCTGCCTACA, Foxp3 REV: GATCCCAGGTGGCAGGC*. Relative quantification was performed based on previously established assay-specific standard curves made with cDNA containing transcripts of the housekeeping gene (β2 M) and the other genes investigated. The relative expression of each target gene was then normalized to the relative expression of β2 M in each sample. Only primers showing comparable efficiency were used. All qPCR reactions were performed in duplicates and average CT was used for relative amount quantification. All qPCR reactions were monitored by melting curve analysis. In all figures containing qPCR data error bars indicate standard deviation amongst biological replicates.

### Antibodies and reagents

The following antibodies (all from Biolegend) were used for cell surface or intracellular staining: Pacific Blue (PB), APC-Cy7 or Alexa Fluor (AF) 700-conjugated anti-TCRβ (H57-597), PB, APC-Cy7-conjugated anti-CD4 (RM4-5), APC-conjugated anti-Thy1.1 (OX-7), PB-conjugated anti-Thy1.2 (53-2.1) PE or APC-conjugated anti-CD44 (IM7), PE or APC-conjugated anti-IL17A (TC11-18H10.1), AF647-conjugated anti-IL17F (9D3.1C8), AF647-conjugated anti-IL17A/F heterodimer (B8KN8R), BV421 or APC-conjugated anti-IFNγ (XMG1.2). APC-conjugated goat anti-mouse Neuropilin-1 (catalog SAB566A) was obtained from R&D systems and APC-conjugated anti-CD25 (PC61), PE or APC-conjugated anti-Foxp3 (FJK-16s), eFluor 450- conjugated antiKi-67 (SolA15), PE–conjugated anti-IL10 (JES5-16E3) from eBiosicences, Inc. Rabbit anti-GFP antibody (catalog 600-402-215 Rockland Immunochemicals) was used to stain Blimp1^*YFP*^ and Foxp3^*GFP*^ reporter cells when intracellular staining for Foxp3 was performed. Zombie Violet™ Fixable Viability Kit (Biolegend) was used for assess live vs. dead status of cells. Foxp3 staining was done using eBioscience Foxp3 staining kit (catalog 42-1403) and BioLegend’s FOXP3 Fix/Perm Buffer Set (catalog #421403). Samples were acquired on a LSRII analyzer (BD Biosciences) and analyzed with FlowJo software (Treestar). For all FACS experiments debris and dead cells were excluded from the analyzed gates.

### Immunoblotting

Immunoblotting were performed with rabbit mAb anti-Blimp1 (clone C14A4; Cell Signaling Technology) and a mouse mAb anti-β-actin (A5441; Sigma-Aldrich). Antibody binding was detected by using SuperSignal West Dura Extended Duration Substrate (Thermo Scientific). Images were acquired on a ChemiDoc XRS (Bio-Rad).

### Intracellular cytokine measurement

Single cell suspension from peripheral organs were stimulated with plate-bound anti-CD3 (5 μg/ml) and anti-CD28 (2.5 μg/ml) for 24 hrs or with PMA (2 ng/ml) and Ionomycin (0.2 ng/ml) for 4 hrs. Brefeldin A was added in the last 6 or 2 hrs of incubation, respectively. Cells were collected, surface stained and fixed with 4% paraformaldehyde followed by intracytoplasmic staining of cytokines.

### Single cell sorting and qPCR

PMA and ionomycin *in vitro*-stimulated CD4^+^ CD25^high^ T cells were single-cell sorted directly into RT-PreAmp Master Mix (Life Technologies). Cell lysis, sequence-specific reverse-transcription and sequence-specific amplification of cDNA were done as previously described^37^ and high-throughput quantitative PCR was done on 96.96 Dynamic Arrays with a BioMark system (Fluidigm San Francisco, CA, USA). Amplification curves were quality filtered-using a threshold of 0.65, and melting curves generated for each reaction were manually inspected as an added quality control step. Cycling threshold (C_T_) values were calculated with BioMark system software for each assay and the same thresholds were used across all experiments. After exclusion of failed reactions, cells in which the expression of at least three different housekeeping genes mRNA was detected were retained for further analysis. All primers (Fluidigm DELTA gene assays) were pre-validate using positive and negative single cells (data not shown). ΔCt values were calculated in reference to the housekeeping genes.

### Histology

At 10–32 wks after birth, mice were sacrificed and colons were removed for histological analysis. Histology samples were stained with H&E and scored by a pathologist in a blinded fashion using the following scoring system: 0=normal; 1=focal inflamamtory cells without ephitelium changes; 2=focal inflmamation w/ ephithelium changes; 3= difuse inflmmmation with epithelium changes/hyperplasia/regeneration/loss of Globet cells; 4= severe difuse inflammation 5= score 4 plus alcerations as previously described.

### In vitro T helper and Foxp3^+^ iTreg differentiation

Naïve CD4^+^ Foxp3^*GFP*−^ CD44^low^ T cells were sorted from spleen and lymph nodes cell suspensions using a FACSAria II fluorescent cell sorter (BD Biosciences). Sorted naïve CD4^+^ T cells were cultured in IMDM with 10% FBS (Omega scientific, Inc) and penicillin/streptomycin (Corning) with plate-bound anti-CD3 (5 μg/ml) and anti-CD28 (2.5 μg/ml). To generate Th1 cells, Naïve CD4^+^ T cells were stimulated with rHuIL-2 (25 U/ml; Roche), IL12 (5 ng/ml; Biolegend) and anti-IL4 antibody (10 μg/ml; BioXcell). For generation of Th17 cells, naïve CD4^+^ T cells were stimulated with IL1β (20 ng/ml; Biolegend), IL23 (50 ng/ml; Biolegend), IL6 (10 ng/ml, Biolegend), anti-IL4 (10 μg/ml), anti-IFNγ antibody (10 μg/ml; BioXcell) and rHuTGF-β1 (5 ng/ml; Biolegend) as previously described^17^. Pathogenic Th17 (pTh17) cells were generated as previously described by Jain *et al*.^18^. Briefly, sorted naïve CD4^+^ T cells were activated with plate-bound anti-CD3 (2.5 mg/ml) and anti-CD28 (1 mg/ml) in the presence of TGF-β (10 ng/ml), IL-6 (100 ng/ml), anti-IFN-γ (10 mg/ml), and anti-IL-4 (10 mg/ml). for 3 days and then rested in the same culture media for another 3 days, followed by re-stimulation with anti-CD3(1 mg/ml) and anti-CD28 (1 mg/ml) in the presence of IL-23 (20 ng/ml) for another 3 days. *In vitro* induced Treg (iTreg) cells were differentiated in the presence of rHuIL-2 (50 U/ml), TGFβ (5 ng/ml) and retinoic acid (10 nM; SIGMA). Expression of Foxp3 in iTreg cultures were usually ≥85% (data not shown) and cells were analyzed in bulk.

### Adoptive transfer of naïve CD4^+^ T cells for generation of Teff and peripheral (p)Treg cells

LNs and spleen were pooled from 8–10 weeks old allotype marked Thy1.1^+^ Ctrl Foxp3GFP reporter mice or Thy1.2^+^ Blimp1CKO CD4^*CRE*^ Foxp3GFP reporter mice. CD4^+^ Naïve (CD4^+^ CD44^low^CD25^−^ Foxp3^*GFP*−^) were sorted and injected (i.p.) on a 1:1 Ctrl/CKO ratio into C57BL/6 RAG1^−/−^ mice (4 × 10^5^ total cells/mouse). Mice were weighed twice a week and inspected for clinical signs of disease (including weight loss, hunched appearance, pilo-erection of fur coat, and loose stool) daily. Mice presenting clinically severe disease (including weight loss, hunched appearance, pilo-erection of fur coat, loose stool and body weight loss equal or greater than 25% of initial body weight) were sacrificed per the CSMC IACUC-approved protocol. At 6–8 wk post-transfer, recipient mice were sacrificed and SP, MLN and LI were isolated for further analysis and/or isolation of CD4^+^ Foxp3^*GFP*+^ pTreg cells and CD4^+^ Foxp3^*GFP*−^ Teff cells.

### RNA Microarray

Total RNA was isolated from sorted (purity ≥ 98%) Ctrl and Blimp1CKOFoxp3^*GFP*+^ pTreg and Foxp3^*GFP*−^ Teff (three biological replicates for each) using Qiagen RNAeasy kits (Qiagen) and RNA quality was assessed with an Agilent Bioanalyzer and a high sensitivity 6000 RNA Pico chip (Agilent, Santa Clara, CA). Only samples with RNA-integrity scores of ≥9.5 were used for further studies. A total of 25 ng of total RNA for each sample was converted and amplified using Ambion’s Illumina Total Prep RNA amplification kit (Austin TX, USA) according to manufacturer’s guidelines and hybridized, washed and labeled on to Illumina MouseWG 6v2 mouse whole-genome bead arrays. For data preprocessing, Illumina Genome Studio software was used to export expression intensity values and the significance of intensity over background signal for each probe of each sample. Genes identified as expressed over background in any two samples were retained for further analysis. Data was normalized by quantile normalization and differential expression analysis was performed by using R package ‘limma’ (v3.30.11). Differential expressed genes with p-value < 0.05 and at least 1.5 fold relative average difference between Ctrl and CKO signals were considered in our comparative analysis of Blimp1-regulated gene expression between Treg and Teff cells. Ingenuity Pathway Analysis (IPA) software was employed to perform comparison analysis of diseases and bio functions (−log10 *p* value cut-off 1.3).

### Statistics

Statistical significance was calculated by Prism (GraphPad Software, Inc., La Jolla, CA). *p* ≤ 0.05 was considered significant. *p* values denoted in figures as follows: ^∗^ *p* < 0.05; ^∗∗^ *p* < 0.01: ^****^ *p* < 0.005

## Electronic supplementary material

Supplementary information
