# Supplementary material for: Differential regulation of Effector and Regulatory T cell function by Blimp1
Source: Sci Rep. 2017 Sep 21;7:12078. doi: 10.1038/s41598-017-12171-3 (PMC5608714; doi:10.1038/s41598-017-12171-3)
Supplement: Supplementary file 1 — Supplementary information [file 41598_2017_12171_MOESM1_ESM.pdf]

Supplementary information

Differential regulation of Effector and Regulatory T cell function by Blimp1

Rashmi Bankot*, Chihiro Ogawa* Truc Nguyen, Lena Emadi, Michael Couse, Soofia Salehi, Xuemo Fan, Deepti Dhall, Yizhou Wang, Jordan Brown, Vincent Funari, Jie Tang, and Gislâine A. Martins

*These authors contributed equally to this work.

SUPPLEMENTARY FIGURE 1

A

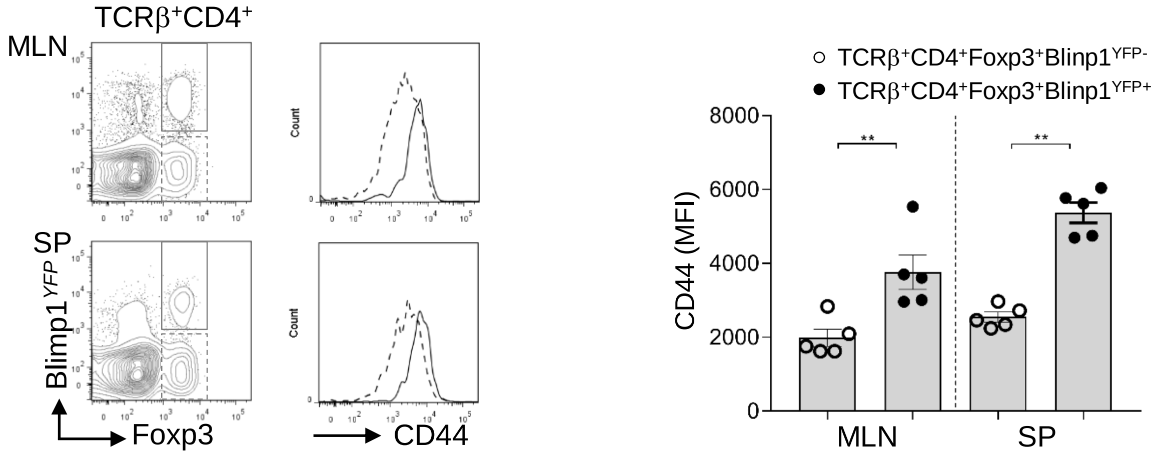

B

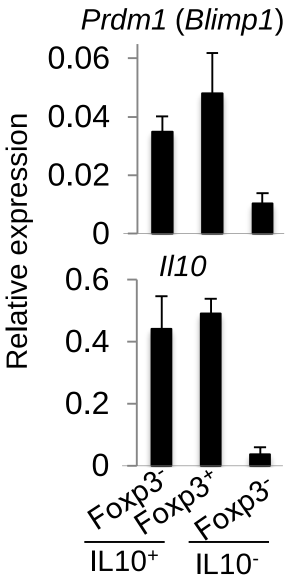

Blimp-1 expression in Treg and Teff cells (**A**) Left, Histograms show CD44 expression in TCRβ$^+$CD4$^+$Foxp3$^+$Blimp^{YFP+} (gate showed in left side FACS plot, solid line) and TCRb$^+$CD4$^+$Foxp3$^+$Blimp^{YFP-} (dashed line) in MLN and SP. Right, bar graph shows average percentage ± SEM of MFI of CD44$^+$ cells in TCRβ$^+$CD4$^+$Foxp3$^+$Blimp^{YFP+} (filled circles) and TCRβ$^+$CD4$^+$Foxp3$^+$Blimp^{YFP-} (open circles) cells in MLN and SP. Each symbol represents one mouse and N=5. (**B**) qRT-PCR analysis of *Prdm1* and *Il10* mRNA in sorted CD4$^+$Foxp3^{GFP-}IL10$^+$ (Tr1), total Foxp3^{GFP+} and Foxp3^{GFP-}IL10$^-$CD4$^+$ T cells from IL10 10Bit-Foxp3^{GFP} mice. (N=3 mice/group). *P<0.05, **P<0.01, paired *t*-test.

SUPPLEMENTARY FIGURE 2

A

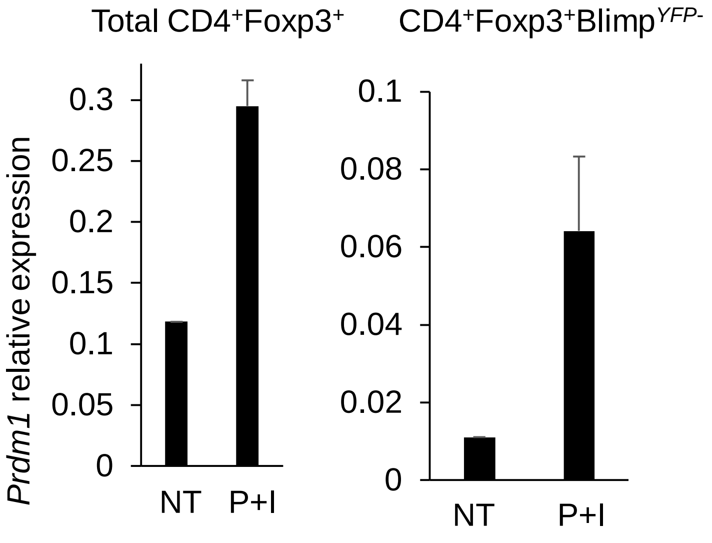

B

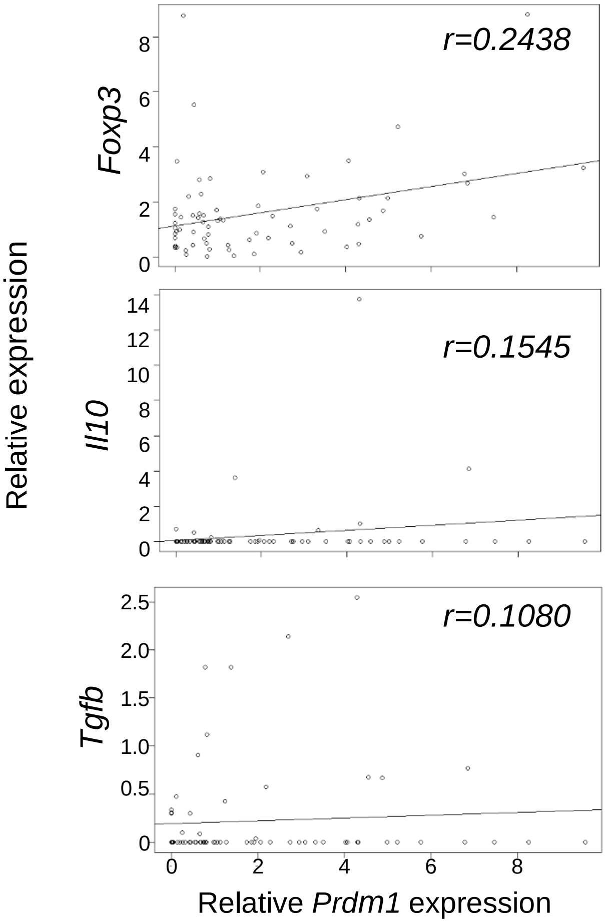

Blimp1 expression is induced upon TCR stimulation in Foxp3^{+} Treg cells but does not correlate with cytokine expression. (**A**) Graphs showing relative expression of *Prdm1* (*Blimp1*) by qRT-PCR in bulk sorted total (CD4^{+} Foxp3^{+}) and Blimp^{YFP-} (Foxp3^{+}Blimp1^{YFP})$^{-}$ Treg cells before (NT) and after in vitro TCR stimulation (4hours, PMA and ionomycin, P+I). (**B**) Graphs showing correlation of *Prdm1* and *Foxp3* (top), *Il10* (middle) or *Tgfb* (bottom) expression in CD4^{+}CD25^{high}T cells. Regression line is presented in each graph. Each symbol represents one cell.

SUPPLEMENTARY FIGURE 3

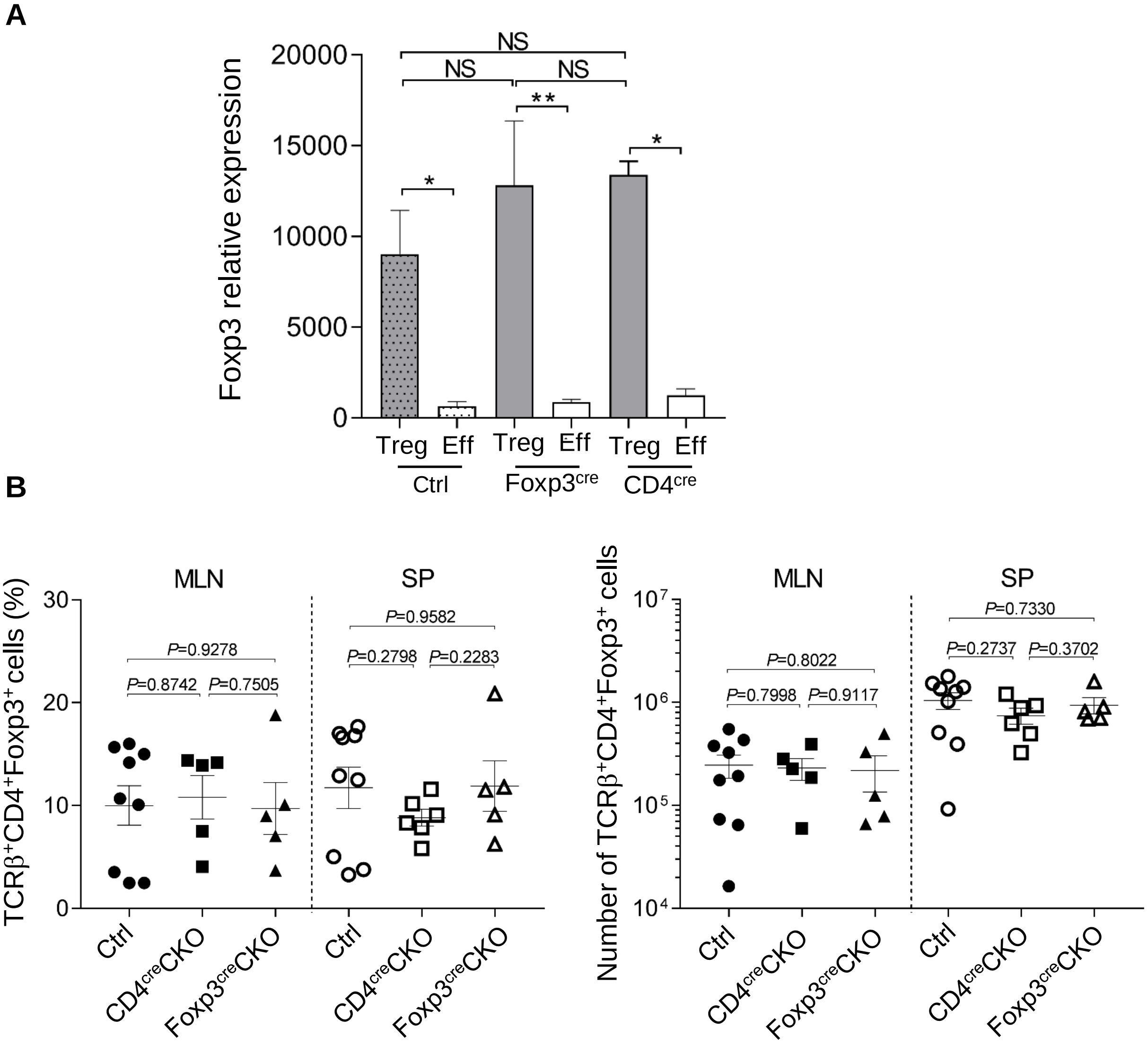

CD4 or Foxp3-driven CRE expression does not alter Foxp3 expression or Foxp3^{+} cell numbers. (A) Expression of Foxp3 mRNA (qRT-PCR, relative to b2Microglobulin) in sorted Treg (CD4^{+} Foxp3^{+}) and Teff (CD4^{+}Foxp3^{-}CD44^{high}) cells from control (*Prdm1*$^{+/+}$), FOXP3^{CRE} CKO (*Prdm1*$^{F/F}$ Foxp3^{cre+}), and CD4^{cre}CKO (*Prdm1*$^{F/F}$ CD4^{cre+}) mice**. (B)** Scatter plots showing the frequency (left) and absolute numbers (right) of TCRβ^{+}CD4^{+}Foxp3^{+} cells in in the mesenteric lymph nodes (MLN) and spleen (SP) from control (Ctrl) and Blimp1 CKO (CD4^{cre}CKO or Foxp3^{cre}CKO) mice. N≥5 mice/group. Bars indicate average ± SEM. *P*-values were calculated by one-way ANOVA.

SUPPLEMENTARY FIGURE 4

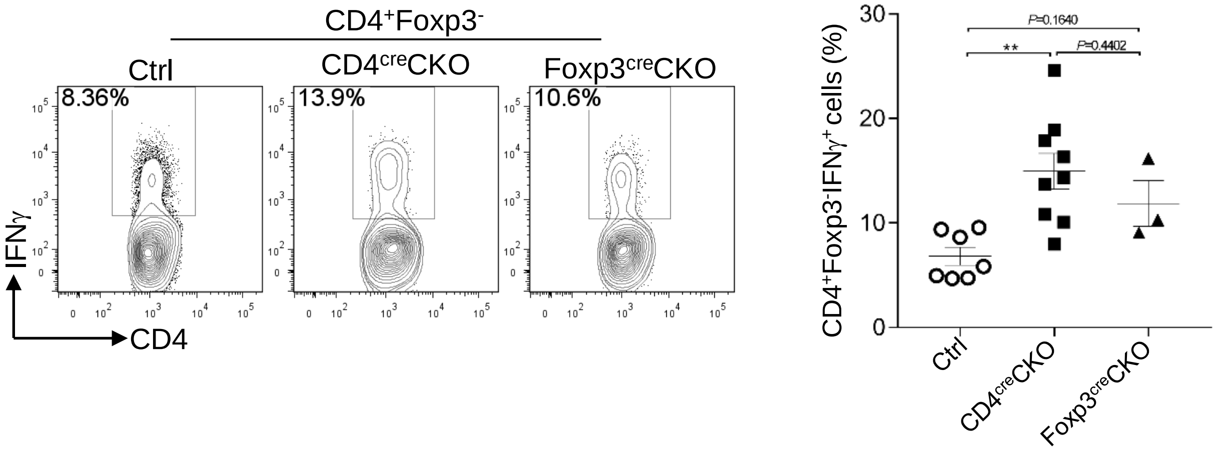

Fox3$^+$Treg cell-specific deletion of Blimp1 does not alter IFNγ production in CD4$^+$Teff cells. FACS plots (left) and scatter plots (right) showing the frequency of IFNγ$^+$CD4$^+$Foxp3$^-$ cells in spleen cells of control (Ctrl) and Blimp1 CKO (CD4^{cre}CKO or Foxp3^{cre}CKO) mice. Cells were stimulated with anti-CD3 and antiCD28 for 24 hours (BFA added in the last 6 hours) prior to staining and analysis. Scatter plot (right): Each symbol represents one mouse (N≥3 mice/group). Bars indicate average ± SEM. ***P*<0.01, one-way ANOVA.

SUPPLEMENTARY TABLE 1. List of Deferentially expressed (DE) genes. DE genes (1.5-fold at *P*<0.05) in pTreg and Teff cells (CD4^{cre}CKO versus Ctrl) generated from Ctrl and CD4^{cre}CKO CD4^{+} naive T cells co-injected into RAG1$^{-/-}$ mice and submitted to mRNA microarray analysis.

Gene	Up/Dowm_pTreg	
	log2FC (KO_pTreg/WT_pTreg)	pvalue
Ccr6	2.520919234	0.0141
Pdlim1	2.273355608	0.0000
Msc	2.220397777	0.0018
Pou2af1	2.049579392	0.0003
Crtam	1.947450066	0.0073
Idi2	1.863956018	0.0005
Il17f	1.794787661	0.0031
Gypc	1.636661509	0.0000
Xcl1	1.624664511	0.0022
Ercc6l2	1.521558802	0.0001
Dnase1l3	1.490428516	0.0045
Slamf7	1.436890232	0.0026
Tespa1	1.41708856	0.0001
Itga3	1.407333133	0.0156
Cd160	1.352369287	0.0308
Bdh2	1.261551864	0.0000
Hif1a	1.253909991	0.0002
Gls2	1.250897919	0.0323
Ssx2ip	1.248955297	0.0000
Sema4f	1.236165115	0.0004
Sigirr	1.231406647	0.0049
Dnph1	1.227720571	0.0006
Gstt1	1.13772866	0.0130
Tnfsf14	1.134561079	0.0252
Lta	1.120098533	0.0007
Impa2	1.119343245	0.0097
Gstt3	1.113026745	0.0155
Gnai1	1.112370983	0.0010
Tnfsf11	1.077909679	0.0012
Pafah1b3	1.056963661	0.0003
Egr4	1.047859531	0.0465
Spsb1	1.02004449	0.0245
Clec2i	1.015977728	0.0273
Tnfrsf26	1.002719996	0.0076
Rhbdf1	0.987815584	0.0279
Gspt2	0.984331923	0.0003
Gramd1b	0.968992393	0.0070
Tcf7	0.960015545	0.0003

Gene		log2FC (KO_pTreg/WT_pTreg)	pvalue
Cxcr3		0.923256362	0.0260
Ece1		0.918057313	0.0056
Cxcr5		0.908289177	0.0092
Kcnn4		0.893542811	0.0342
Ccdc50		0.885353084	0.0001
Capn5		0.871089256	0.0010
Ankrd46		0.86157782	0.0054
Kras		0.858361977	0.0015
Stat4		0.851729084	0.0018
Jazf1		0.847377688	0.0364
Cep85		0.82999805	0.0001
D030056L22Rik		0.82242731	0.0008
Pax1		0.806027649	0.0117
Adh4		0.799159123	0.0025
Unc13b		0.788993399	0.0415
Arl6		0.777257241	0.0011
Txnrd3		0.768740902	0.0001
Slc16a6		0.766421394	0.0002
Lrmp		0.765402822	0.0227
Rbbp7		0.762115907	0.0333
Bcl2		0.761068908	0.0000
Ptk2		0.758203054	0.0383
Zdhhc13		0.755337823	0.0002
Wisp1		0.75261012	0.0037
Pgam1		0.752045434	0.0002
Zfp202		0.748903283	0.0019
Crip2		0.74630009	0.0043
Slc29a3		0.742670565	0.0165
Tpi1		0.729284155	0.0263
Tmem41a		0.726381025	0.0143
Utf1		0.720474045	0.0196
1110038B12Rik		0.720236036	0.0193
Plac8		0.719005765	0.0386
Sdf4		0.716346108	0.0022
Tg		0.713552254	0.0018
Ggt7		0.712584294	0.0054
Zfp280b		0.711268672	0.0156
Ptprk		0.706691942	0.0349
Hvcn1		0.705710168	0.0218
Prelid2		0.70017582	0.0097
Pus7l		0.696389705	0.0079
Tmem216		0.693784923	0.0080

Cdon	0.68891227	0.0061
2310001H17Rik	0.688070435	0.0187
Pdzk1	0.684307044	0.0049
Xpot	0.680222684	0.0269
Zfp940	0.678521027	0.0008
Krt17	0.662150809	0.0221
Anp32a	0.660326989	0.0016
Trps1	0.657515751	0.0166
Sccpdh	0.654549408	0.0020
Ccdc64	0.651771459	0.0231
Tmed4	0.644714636	0.0160
Wrb	0.644040647	0.0103
Nudt5	0.642985532	0.0240
Ttc27	0.638604467	0.0051
Cd320	0.631470764	0.0092
Abca3	0.619945219	0.0055
Serpind1	0.613884693	0.0446
Rcan1	0.612675005	0.0103
Il11	0.610745451	0.0029
Tspan2	0.609770854	0.0106
Itpr1	0.609407357	0.0281
Tspan3	0.608915137	0.0054
Slc24a3	0.608579103	0.0402
Trpm6	0.607082471	0.0040
Acaca	0.603891458	0.0056
Hspd1	0.600697468	0.0036
Mid2	0.598074879	0.0021
0610037L13Rik	0.595253542	0.0151
Snhg5	0.593638313	0.0003
Fam69b	0.590580045	0.0027
Ybx3	0.587527173	0.0010
Als2	0.585691642	0.0093
Irf1	-0.585076974	0.0016
Parp14	-0.587108183	0.0004
Sh3bgrl3	-0.589111206	0.0117
Capg	-0.590601888	0.0481
Neurl3	-0.591404438	0.0049
Sash3	-0.593500551	0.0158
Ubl3	-0.593632561	0.0007
Dctn6	-0.59368767	0.0076
Tnfrsf4	-0.594253288	0.0031
Ly6a	-0.596764506	0.0085
F2rl2	-0.599186669	0.0070
Rab4b	-0.601522963	0.0003
Coro1a	-0.603454273	0.0150
Fam177a	-0.603726894	0.0407
Map4k2	-0.60448233	0.0004
Stk38l	-0.605170484	0.0032
Tor4a	-0.608702599	0.0117
Cryba4	-0.611502727	0.0117
Oaz2	-0.612393257	0.0008
4632428N05Rik	-0.613260283	0.0219
Egr1	-0.613370611	0.0498
Grb7	-0.615017239	0.0104
Ppm1l	-0.618120832	0.0153
Jun	-0.619222454	0.0347
Zfp281	-0.619981476	0.0203
Fli1	-0.621015115	0.0057
Tspan14	-0.621233385	0.0073
Plekho2	-0.624023499	0.0247
Arrdc4	-0.624216062	0.0399
Ppp1r12a	-0.624377812	0.0367
Zmynd8	-0.6256919	0.0107
Armc7	-0.628397526	0.0347
Ikzf4	-0.629169147	0.0113
Samsn1	-0.630887968	0.0101
Apaf1	-0.634243863	0.0217
Slc9a3r1	-0.635494307	0.0017
Mif4gd	-0.638467848	0.0183
Snx20	-0.641606582	0.0122
Sorl1	-0.641715352	0.0063
Foxj2	-0.643976935	0.0113
Kmt2a	-0.645931795	0.0080
Cggbp1	-0.646219223	0.0079
Ddit3	-0.646354393	0.0032
Icos	-0.647691685	0.0219
Arhgap18	-0.650046033	0.0292
Agfg1	-0.654665642	0.0076
Ak7	-0.656181329	0.0201
Tbx21	-0.656652127	0.0301
Commd3	-0.656725294	0.0094
Cyth1	-0.65780697	0.0079
Eci2	-0.658905414	0.0015
3300005D01Rik	-0.662142307	0.0339
Vopp1	-0.664491959	0.0003
Gng12	-0.665226369	0.0021

**Lst1**	-0.669555418	0.0136
**Trex1**	-0.669653835	0.0068
**Zfp263**	-0.671406486	0.0063
**Ppp1r3f**	-0.676308023	0.0107
**Cd53**	-0.676955513	0.0111
**Klc3**	-0.678412845	0.0031
**Ccs**	-0.678767096	0.0492
**S100a10**	-0.680524775	0.0464
**Pigx**	-0.683971636	0.0362
**Acsbg1**	-0.686581561	0.0385
**Irgm2**	-0.687411307	0.0020
**Folr4**	-0.688273953	0.0050
**Lasp1**	-0.689524587	0.0079
**Myo1g**	-0.694030235	0.0227
**Txnip**	-0.696763321	0.0074
**Ehd1**	-0.699656526	0.0028
**Cdkn1a**	-0.705926343	0.0189
**Abi3**	-0.706682188	0.0053
**Samd9l**	-0.708605026	0.0005
**Smim14**	-0.711025506	0.0007
**Cd8b1**	-0.711690115	0.0088
**Lrrc8c**	-0.713941043	0.0067
**Mknk2**	-0.71438017	0.0007
**Plk2**	-0.716729559	0.0076
**Mpp1**	-0.717943712	0.0327
**Hcls1**	-0.718113944	0.0032
**Fam126a**	-0.721185924	0.0203
**Hdac7**	-0.725636174	0.0077
**Tecpr1**	-0.727864921	0.0010
**Rab7l1**	-0.728352222	0.0019
**Cyp4v3**	-0.728463569	0.0079
**Cyb5**	-0.731900961	0.0012
**Ifi47**	-0.735273585	0.0120
**Prkca**	-0.737917472	0.0146
**Ypel3**	-0.737989488	0.0101
**Ccdc92**	-0.740171312	0.0035
**Picalm**	-0.740528917	0.0004
**Rnf213**	-0.743504004	0.0038
**Nuak2**	-0.744360011	0.0189
**Rin3**	-0.746892211	0.0080
**C78339**	-0.747465641	0.0007
**Bmp7**	-0.747897474	0.0051
**Agpat4**	-0.751347981	0.0002

**Lyrm5**	-0.753095049	0.0000
**Jakmip1**	-0.75387727	0.0000
**Pglyrp1**	-0.762341864	0.0189
**Irak3**	-0.763465674	0.0279
**Aes**	-0.765956687	0.0002
**Dtx1**	-0.766604237	0.0069
**Rasal3**	-0.770531704	0.0004
**Ebpl**	-0.772263856	0.0129
**Hexa**	-0.773257869	0.0048
**Stk17b**	-0.774665861	0.0080
**Gpr146**	-0.774873737	0.0004
**Atf7ip**	-0.778440597	0.0259
**Nck2**	-0.779290082	0.0049
**Rab32**	-0.78262	0.0016
**Mta3**	-0.789378396	0.0018
**Src**	-0.790023207	0.0074
**Jak1**	-0.79193387	0.0116
**Pdgfb**	-0.797587855	0.0181
**2210408F21 Rik**	-0.798862313	0.0058
**Lipa**	-0.800189619	0.0100
**Mob3a**	-0.802463619	0.0002
**Upp1**	-0.807019926	0.0039
**Maf**	-0.810529808	0.0006
**Rgs10**	-0.818593834	0.0274
**Ppp1r18**	-0.819170258	0.0001
**AI467606**	-0.822235189	0.0027
**Ech1**	-0.82251202	0.0010
**Gbp2b**	-0.823726097	0.0316
**Ptms**	-0.826632887	0.0112
**Kansl1l**	-0.827342834	0.0011
**Inpp5d**	-0.831815406	0.0011
**Fam134b**	-0.831830112	0.0123
**Apobec1**	-0.832189123	0.0175
**Dok2**	-0.834839722	0.0003
**Anxa6**	-0.841781898	0.0015
**Cxcl2**	-0.842540498	0.0070
**Dhrs1**	-0.843255117	0.0037
**Uba7**	-0.846420478	0.0006
**Rac2**	-0.854198135	0.0019
**Npc2**	-0.875220256	0.0055
**Swap70**	-0.875251493	0.0360
**Mmp13**	-0.879650559	0.0005
**Klf2**	-0.880763631	0.0289

**Ank**	**-0.882409732**	**0.0027**
Stk32c	-0.883017082	0.0014
Abcb9	-0.884132588	0.0022
Gm4951	-0.8863652	0.0082
Slfn1	-0.891874237	0.0149
Sat1	-0.898998323	0.0103
F2r	-0.900274643	0.0072
Tdrd7	-0.903459804	0.0002
Oas1g	-0.905979159	0.0106
Gadd45a	-0.90723354	0.0030
Gbp3	-0.908435957	0.0065
Endod1	-0.922761334	0.0014
Xaf1	-0.943697141	0.0070
Hmha1	-0.944461496	0.0001
Rbms1	-0.948557565	0.0000
Igtp	-0.949839755	0.0002
Sema4a	-0.970895275	0.0041
S100a13	-0.982292821	0.0148
Spata13	-0.988544718	0.0016
Cdkn2b	-0.992484895	0.0055
Ky	-0.995461824	0.0000
Gbp5	-1.002381393	0.0088
N4bp1	-1.005486756	0.0000
Emc9	-1.00722179	0.0016
Itk	-1.010389033	0.0104
Gng2	-1.015432571	0.0186
Serpinb6a	-1.016418438	0.0093
Sgms1	-1.019926255	0.0008
Gimap6	-1.020885862	0.0006
Csrp2	-1.028290796	0.0005
Il6st	-1.028351235	0.0092
Ppp1r3b	-1.048608337	0.0000
Icam1	-1.048891816	0.0003
Pea15a	-1.049359032	0.0006
Hsd11b1	-1.051909481	0.0032
Marcks	-1.054384773	0.0065
Nkg7	-1.054761587	0.0368
Syp	-1.064701299	0.0167
Camk2n1	-1.095658603	0.0098
Sgk1	-1.096984078	0.0034
Ctla4	-1.104960289	0.0006
Bcl3	-1.105714901	0.0059
Selplg	-1.108699562	0.0001

Ifitm3	-1.133460529	0.0180
Tnfrsf18	-1.161189574	0.0007
Pdzk1ip1	-1.167085364	0.0367
Ecm1	-1.170183991	0.0012
Il13	-1.174240918	0.0087
Glipr1	-1.174447465	0.0003
Dgat1	-1.177746651	0.0076
Fam132a	-1.181717716	0.0021
Gna15	-1.184325034	0.0058
Lxn	-1.191274969	0.0001
Arhgap4	-1.20463949	0.0000
Ccl4	-1.221322256	0.0005
Gm4956	-1.223012066	0.0007
Myo1f	-1.225138307	0.0081
Gimap7	-1.228687702	0.0103
Ifngr1	-1.254746189	0.0035
Slc2a3	-1.25896302	0.0016
Arl4c	-1.262381331	0.0103
Trpv2	-1.272370057	0.0013
Zfp36	-1.292528664	0.0001
Ikzf2	-1.334553701	0.0138
Ltb4r1	-1.455145237	0.0005
Foxp1	-1.472045464	0.0008
Cd72	-1.571580275	0.0177
Isg20	-1.665804876	0.0002
Socs2	-1.79608408	0.0002
Glrx	-1.844945755	0.0000
Cytip	-1.933855188	0.0013
Snx9	-1.972830927	0.0007
Il10ra	-2.099839132	0.0000
Gpr83	-2.211314476	0.0000
Ccr5	-2.250752091	0.0002
Ncmap	-2.804431998	0.0001

Gene	Up/Dowm_T effector	
	log2FC (KO_Teff/WT _Teff)	pvalue
**Prss34**	5.172263149	0.0314
**Mcpt8**	4.413352029	0.0164
**Ccl9**	4.04151651	0.0082
**Il6**	3.505771403	0.0221
**Cpa3**	3.232991333	0.0209
**Cyp11a1**	3.227220802	0.0155
**Lat2**	**3.10096442**	**0.0173**
**Gja1**	2.986859818	0.0348
**Il1b**	2.870422519	0.0455
**H2-Eb1**	2.768839733	0.0263
**Fcer1a**	2.766447424	0.0202
**Aqp9**	2.75662276	0.0199
**Cd63**	2.693807736	0.0256
**Klre1**	2.689236897	0.0240
**Hs3st1**	2.536952403	0.0217
**Osr2**	2.505617017	0.0058
**Napsa**	2.489581795	0.0143
**Adora2b**	2.474845611	0.0294
**Klra18**	2.43120066	0.0464
**H2-Ab1**	2.378837445	0.0171
**Tgm1**	2.369254697	0.0301
**Pdzk1ip1**	2.36191606	0.0063
**H2-Aa**	2.328800707	0.0074
**Csf1**	2.317876659	0.0153
**Klra3**	2.311397338	0.0462
**Trf**	2.068943646	0.0314
**Inhba**	2.047635797	0.0444
**Cd83**	2.014026276	0.0157
**Scimp**	2.000053962	0.0404
**Cd74**	1.843380308	0.0128
**Klf5**	1.747992377	0.0489
**Itga2b**	1.678158762	0.0343
**Armcx2**	1.598319056	0.0023
**Cd200r3**	1.50893154	0.0240
**Tubb6**	1.470263131	0.0359
**Fndc3b**	1.45960737	0.0049
**Ccdc22**	1.453768055	0.0311
**Cd9**	1.423679976	0.0189
**Swap70**	1.406612408	0.0072
**Csf2rb2**	1.374044556	0.0114
**Tnfsf4**	1.352674824	0.0009
**Fam110c**	1.347288324	0.0331
**Car2**	1.339165821	0.0246
**Pcyt1a**	1.321013247	0.0223
**Cxcr4**	1.320803523	0.0024
**Ifitm1**	1.317555602	0.0058
[illegible]	[illegible]	[illegible]

Gene		
	log2FC (KO_Teff/WT _Teff)	pvalue
**Tacstd2**	**1.222627115**	**0.0136**
**Car13**	1.179831358	0.0272
**Abcd3**	1.172626551	0.0177
**Aqp3**	1.138448259	0.0482
**Cx3cl1**	1.103867942	0.0001
**Lrig1**	1.102030796	0.0469
**H2-DMb1**	1.063444554	0.0456
**Ppap2c**	1.056348346	0.0257
**Rap2a**	1.035345158	0.0170
**Gns**	1.012801425	0.0106
**Klrk1**	1.010575951	0.0273
**Slco2b1**	0.998175655	0.0185
**Cd81**	0.990650833	0.0287
**Nucb1**	0.981372047	0.0233
**Ttc7b**	0.964754972	0.0338
**Tax1bp1**	0.959886663	0.0257
**Orai3**	0.953848719	0.0091
**Plcg2**	0.951444416	0.0121
**Gpr183**	0.922500427	0.0332
**Gzmm**	0.903082682	0.0126
**Snx8**	0.885686956	0.0253
**Sav1**	0.861381282	0.0057
**Igfbp4**	0.850449101	0.0275
**Nt5dc2**	0.847923369	0.0222
**Fosl1**	0.836776991	0.0231
**Chpf**	0.832768897	0.0109
**Mlkl**	0.832083144	0.0056
**Prkd3**	0.82856238	0.0255
**Stx7**	0.813740066	0.0376
**Trio**	0.805316734	0.0352
**Cerk**	0.795965962	0.0129
**Myo6**	0.7936571	0.0076
**Ctsc**	0.7792256	0.0244
**Large**	0.740962027	0.0071
**Amd1**	0.737331547	0.0002
**Gaa**	0.731173427	0.0220
**Tcf4**	0.73030842	0.0022
**Tiam2**	0.729669788	0.0245
**Ccnd2**	0.71645197	0.0414
**Casz1**	0.708629453	0.0157
**Rps4y2**	0.699184242	0.0137
**Avpi1**	0.698268597	0.0241
**Wasf2**	0.69211529	0.0339

**Mfge8**	**0.688784502**	**0.0130**
**Cgrrf1**	0.675455508	0.0041
**Fam109a**	0.673705083	0.0054
**Sirt3**	0.664131545	0.0363
**Ftl1**	0.659238418	0.0157
**Zfp219**	0.658278875	0.0077
**Cd47**	0.658173809	0.0243
**Golim4**	0.653352536	0.0041
**Pgd**	0.651480541	0.0044
**Cryba4**	0.633866744	0.0390
**Prodh**	0.622760841	0.0146
**Cdkn1c**	0.621242845	0.0100
**Avil**	0.61822728	0.0215
**Vkorc1**	0.616325834	0.0100
**Tmed1**	0.615861129	0.0270
**Dgkd**	0.613382572	0.0020
**2310044G17Rik**	0.611226058	0.0479
**Ccl6**	0.609642631	0.0327
**Tuba8**	0.604274365	0.0349
**Plxnd1**	0.590704647	0.0299
**Mgat4b**	0.58871331	0.0017
**Klk8**	-0.593556843	0.0365
**Card10**	-0.596561084	0.0205
**Cd96**	-0.596998019	0.0303
**Fam160a2**	-0.609895783	0.0111
**Cd28**	-0.611667462	0.0049
**Olfr802**	-0.615662516	0.0376
**Chchd2**	-0.619764359	0.0034
**Slc12a7**	-0.624931273	0.0108
**Ccng2**	-0.637040883	0.0051
**Slc25a47**	-0.63708513	0.0011
**Cd6**	-0.639403865	0.0132
**Batf**	-0.639688622	0.0303
**Prkcq**	-0.658938549	0.0422
**Slc29a1**	-0.684874498	0.0050
**Spns1**	-0.684997284	0.0338
**Slc25a19**	-0.685921291	0.0039
**Slamf1**	-0.717853123	0.0470
**Amn**	-0.725268584	0.0095
**Tiam1**	-0.731739745	0.0044
**Il12rb1**	-0.738136652	0.0486
**Ext1**	-0.743630571	0.0082
**Bcl11b**	-0.75581781	0.0416
**St6galnac2**	**-0.764351549**	**0.0269**
**Tmc6**	-0.767018294	0.0071
**Pou2f2**	-0.799779423	0.0235
**Ccno**	-0.805836987	0.0271
**Mapre2**	-0.811385404	0.0170
**Ccl20**	-0.831443811	0.0372
**Il17a**	-0.850382287	0.0042
**B4galnt4**	-0.874927182	0.0368
**Rftn1**	-0.902460468	0.0009
**Il22**	-0.914070842	0.0160
**Rnf166**	-0.938472578	0.0163
**Tmem194b**	-0.94503979	0.0015
**Zc3h12d**	-0.950650273	0.0320
**Zfp862**	-1.009466739	0.0161
**Adam19**	-1.057416873	0.0008
**Ltb**	-1.121609677	0.0272
**Sytl1**	-1.136113676	0.0015
**Ubash3a**	-1.172598063	0.0471
**Asb2**	-1.231304664	0.0020
**Sit1**	-1.247730618	0.0038
**Rorc**	-1.336381098	0.0010
**1700012B07Rik**	-1.54432167	0.0044
**Snai3**	-1.618890182	0.0091
**Gcg**	-2.065497725	0.0473

Gene	log2FC (KO_pTreg/WT_pTreg)	pvalue
**Lif**	2.435714619	0.0007
**Slc2a6**	2.727399534	0.0000
**Tgm2**	1.313851509	0.0234
**Prdm1**	1.587528301	0.0001
**Batf3**	2.416833906	0.0007
**Dennd3**	0.766024008	0.0100
**Synpo**	1.55167614	0.0001
**Lhfpl2**	1.743330727	0.0056
**Pecam1**	0.656640532	0.0259
**Bcl6**	1.796873568	0.0001
**1700025G04Rik**	1.553317849	0.0003
**Maged2**	1.720901061	0.0008
**Vcl**	0.721689183	0.0268
**Flnb**	0.980864799	0.0002
**D930015E06Rik**	0.800969652	0.0027
**Tagap**	1.094227684	0.0062
**Tox**	1.068661756	0.0069
**Hspa2**	0.737391271	0.0142
**Anxa2**	0.64288339	0.0074
**Ly6g5b**	-0.725726242	0.0004
**Fam102a**	-0.628043794	0.0145
**Pkp3**	-0.802461518	0.0013
**Trib2**	-0.652171265	0.0029
**Grap2**	-0.664435542	0.0003
**Rasl11a**	-0.93158291	0.0033
**BC021614**	-0.930663925	0.0040
**Malt1**	-0.893780483	0.0161
**Lat**	-0.665501591	0.0030
**Cd5**	-0.636784791	0.0003
**Cpd**	-0.903755402	0.0265
**Stambpl1**	-1.075625487	0.0009
**Rab27a**	-1.134068221	0.0003
**Ckb**	-1.130171493	0.0029
**Notch1**	-1.00169425	0.0028
**A430093F15Rik**	-1.199290105	0.0001
**Tmem154**	-1.245325106	0.0038
**Il10**	-5.199474885	0.0000
